# Six Autoimmune Disorders Are Associated With Increased Incidence of Gastric Cancer: A Systematic Review and Meta-Analysis of Half a Million Patients

**DOI:** 10.3389/fimmu.2021.750533

**Published:** 2021-11-23

**Authors:** Noémi Zádori, Lajos Szakó, Szilárd Váncsa, Nóra Vörhendi, Eduard Oštarijaš, Szabolcs Kiss, Levente Frim, Péter Hegyi, József Czimmer

**Affiliations:** ^1^ Institute for Translational Medicine, Medical School, University of Pécs, Pécs, Hungary; ^2^ János Szentágothai Research Centre, University of Pécs, Pécs, Hungary; ^3^ Centre for Translational Medicine, Semmelweis University, Budapest, Hungary; ^4^ Doctoral School of Clinical Medicine, University of Szeged, Szeged, Hungary; ^5^ Heim Pál National Pediatric Institute, Budapest, Hungary; ^6^ Division of Pancreatic Diseases, Heart and Vascular Center, Semmelweis University, Budapest, Hungary; ^7^ Division of Gastroenterology, First Department of Medicine, Medical School, University of Pécs, Pécs, Hungary

**Keywords:** : autoimmune disease, gastric cancer, autoimmunity, risk, standardized incidence rate

## Abstract

**Background:**

Gastric cancer is one of the most common cancers worldwide, with a high mortality rate. The potential etiological role of autoimmune (AI) disorders has been described in gastric cancer; however, the literature is controversial. This study aims to provide a comprehensive summary of the association between autoimmune disorders and the incidence of gastric cancer.

**Methods:**

This study was registered on PROSPERO under registration number CRD42021262875. The systematic literature search was conducted in four scientific databases up to May 17, 2021. Studies that reported standardized incidence rate (SIR) of gastric cancer in autoimmune disorders were eligible. We calculated pooled SIRs with 95% confidence intervals (CIs) in this meta-analysis.

**Results:**

We included 43 articles describing 36 AI disorders with data of 499,427 patients from four continents in our systematic review and meta-analysis. Significantly increased incidence of gastric cancer was observed in dermatomyositis (SIR = 3.71; CI: 2.04, 6.75), pernicious anemia (SIR = 3.28; CI: 2.71, 3.96), inflammatory myopathies (SIR = 2.68; CI:1.40; 5.12), systemic lupus erythematosus (SIR = 1.48; CI: 1.09, 2.01), diabetes mellitus type I (SIR = 1.29; CI:1.14, 1,47), and Graves’ disease (SIR = 1.28; CI: 1.16, 1.41). No significant associations could be found regarding other AI disorders.

**Conclusions:**

Pernicious anemia, Graves’ disease, dermatomyositis, diabetes mellitus type I, inflammatory myopathies, and systemic lupus erythematosus are associated with higher incidence rates of gastric cancer. Therefore, close gastroenterological follow-up or routinely performed gastroscopy and application of other diagnostic measures may be cost-effective and clinically helpful for patients diagnosed with these autoimmune diseases.

## Introduction

Malignant neoplasm of the stomach is one of the most common cancers worldwide, affecting over 20,000 patients yearly in the USA. The average 5-year survival rate is less than 20%, underlining the importance of the disease (1, 2). This poor prognosis can be improved by early diagnosis. If the tumor is detected and treated before reaching the muscular layer of the stomach (T1), the 5-year survival rate can be up to 90% (3).

A significant decline in incidence and mortality can be observed over the past few decades (4), which can be attributed to the recognition of certain causative factors, decreased incidence of *Helicobacter pylori* infection, and decreased use of tobacco and dietary salt (2, 5). While the overall rate of gastric cancer has been declining, the distribution of its subtype was changing neoplasms of the cardia and gastro-esophagal junction became more frequent, and an unexplained increased incidence among younger than 50 years of age, particularly in females, could be observed (5–8).

Despite the effective *H. pylori* eradication strategies, gastric cancer remains the fifth most common cancer worldwide (9), highlighting the possibility of further etiological factors. Besides *H. pylori*, autoimmune gastritis is another common cause of gastric cancer, reflecting 7.8%–19.5% of the cases, and thought to be another possible cause of the rising incidence of gastric cancer in females younger than 50 years of age (5, 7, 10).

The incidence of autoimmune gastritis and generally autoimmune diseases has increased in the past few decades (11–13). Several previous studies have described the potential association of autoimmunity and gastric cancer (14, 15), but up to date, data have been controversial regarding cause-effect relationships and underlying pathomechanism. Our study aims to provide a comprehensive summary of the potential association between autoimmune disorders and the incidence of gastric cancer in the form of a meta-analysis and systematic review.

## Methods and Materials

This meta-analysis was conducted following the Preferred Reporting Items for Systematic Reviews and Meta-Analyses (PRISMA) 2020 Statement (16). The protocol of this analysis was registered on the PROSPERO International Prospective Register of Systematic Reviews in advance (CRD42021262875). We did not deviate from the protocol.

### Systematic Search

The systematic literature search was conducted in four scientific databases—MEDLINE *via* PubMed; Cochrane Central Register of Controlled Trials (CENTRAL); Embase; and Web of Science, Latin American and Caribbean Health Sciences Literature (LILACS)—up to May 17, 2021. The following search terms were used without any restriction to language or other filters: (stomach OR gastric) AND (neoplas* OR malign* OR cancer OR carcinoma OR lymphoma OR tumor OR tumour) AND (“autoimmun*” OR autoaggressive OR autoantibody OR lupus OR rheuma* OR Addison* OR celiac OR “gluten sensitive” OR dermatomyositis OR Hashimoto OR graves OR sclerosis OR scleroderma OR myasthenia OR arthritis OR Sjögren*). Additionally, reference lists of the citing and cited articles were screened for eligibility.

### Selection and Eligibility of Studies

Duplicates were removed with EndNote X9 software (Clarivate Analytics, Philadelphia, PA, USA) manually. Two investigators (NV, NZ) screened the titles and abstracts and full texts to identify eligible articles. Disagreements were resolved by another investigator (LF, JC).

We included any peer-reviewed studies reporting the standardized incidence ratio (SIR) *(O)* of gastric cancer in an autoimmune disorder *(E)* in the general population (P). There were no restrictions on the type of gastric cancer, language, or study design eligible for inclusion. Only full texts were included. Studies with no event rate of SIR were excluded.

### Data Extraction

Two independent researchers (NZ, NV) extracted data from the eligible studies into a standardized data collection form. Extracted data were validated by a third reviewer (LF). All disagreements were resolved by a fourth independent author (SV). The following data were extracted from each included study: title, first author, year of publication, country, study design, age of the population (mean, standard deviation (SD), median, interquartile ranges), gender distribution, the total number of patients (with autoimmune disorders), type of autoimmune disorders, follow-up time, and standardized incidence ratios of gastric cancer (observed, expected, SIR, confidence interval).

### Data Synthesis

We provided summaries of the rate of gastric cancer in each autoimmune disorder (frequency of gastric cancer in each autoimmune disease) by pooling standardized incidence ratios (SIRs) as an outcome for selected autoimmune disorders. SIRs were first extracted and then pooled using the inverse variance method and random-effects model with the restricted maximum-likelihood (REML) estimation. Subsequently, the results were displayed on forest plots. Summary SIR estimation, *p*-value, and 95% confidence interval (CI) were calculated.

Statistical heterogeneity was analyzed using the *I*² statistic and the *χ*² test to acquire probability values; *p* < 0.1 is defined to indicate significant heterogeneity. As suggested by the Cochrane Handbook, *I* ^2^ values were interpreted as moderate (30%–60%), substantial (50%–90%), and considerable (75%–100%) heterogeneity (17). Publication bias was checked by Funnel plot and Egger’s test (alpha = 0·1) (18). The Eggers test was performed for each autoimmune disorder, where there were more than 10 studies included.

Subgroup analyzes were performed considering high-incidence or low-incidence countries for gastric cancer (10) and based on gender. A minimum number of studies were three for performing quantitative synthesis. Otherwise, findings were summarized in the qualitative synthesis.

All analyses were performed using R statistical software (R Foundation, Vienna, Austria) with the meta package (Guido Schwarzer, v4.18-2).

### Risk of Bias Assessment in Individual Studies

Based on the recommendations of Cochrane Prognosis Methods Group (PMG), the Quality in Prognostic Studies (QUIPS) tool was used by two independent investigators (NV, LF) to assess the quality of the studies included, focusing on the definition of prognostic factors and outcomes (19). Disagreements were resolved by a third investigator (NZ). Details of the QUIPS are shown in **Supplementary Table S2**.

## Results

### Search and Selection

**Figure 1:**
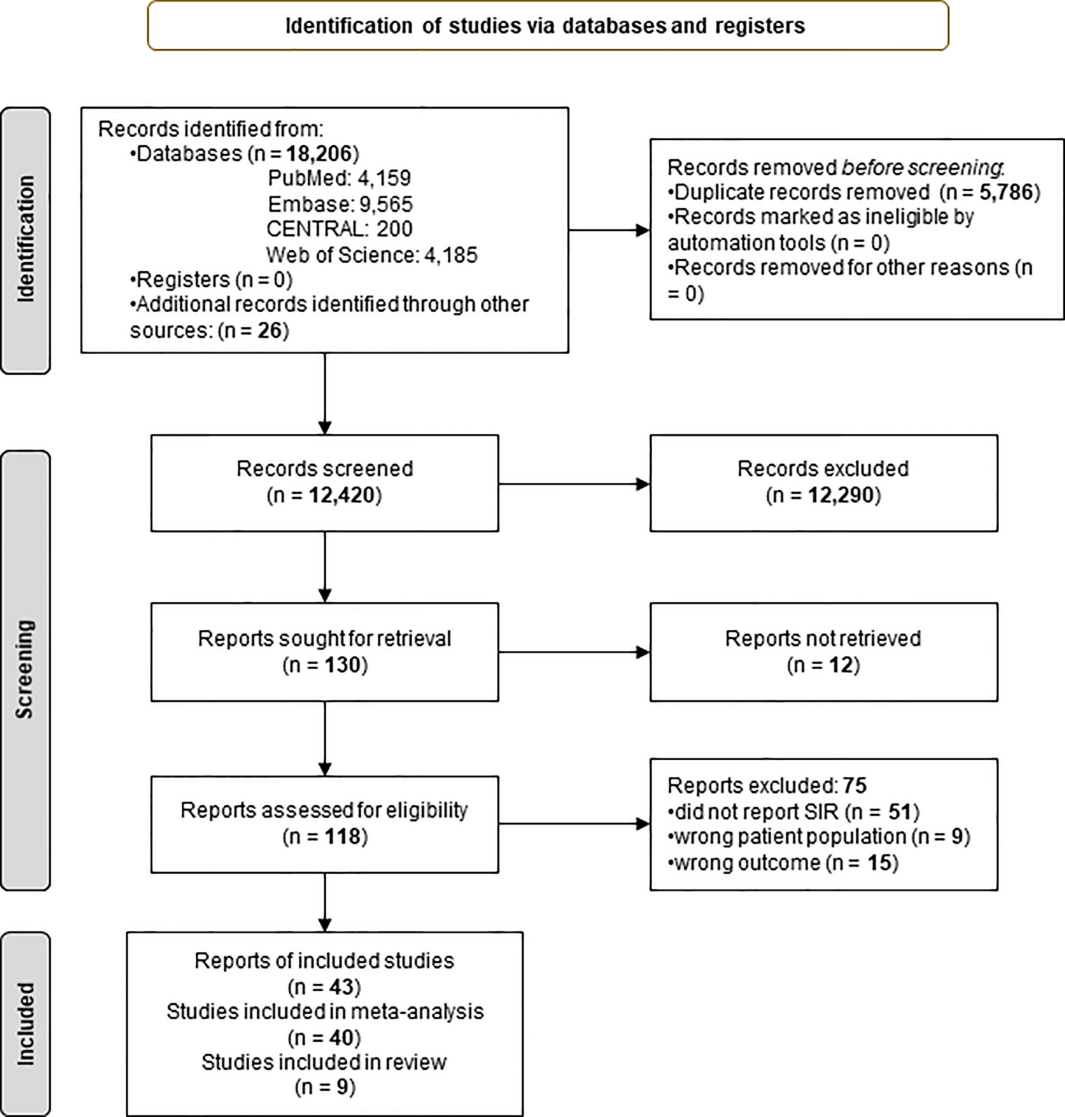
Preferred Reporting in Systematic Reviews and Meta-analyses 2020 (PRISMA) flowchart showing the selection process (16).

The systemic search yielded 18,206 records, of which 12,420 remained after duplicate removal. Following the selection process, 43 articles were included in the systematic review and meta-analysis. Results of the selection are presented in **Figure 1**.

### Basic Characteristics of the Included Studies

Four studies were retrospective from the included 43 articles, and 39 were prospective, describing 36 autoimmune disorders altogether. The overall work, including the qualitative and quantitative synthesis, contains 499,427 patients from four continents (America, Europe, Asia, and Australia) and 15 countries. The general characteristics of the included articles are presented in **Table 1**.

**Table 1:** Basic characteristics of included studies.

Author	Year	Country	Disease(s) Studied	Study Population (% of females)	SIR of Gastric Cancer (95% CI)
Asano et al.	2015	Japan	AIP	109 (23)	1.35 (0.03–2.66)
			IgG4-RD	158 (25)	1.43 (0.03–2.83)
Askling et al.	2002	Japan	Celiac disease	11,019 (59)	0.90 (0.3–2.0)
			Dermatitis herpetiformis	1,354 (43)	1.4 (0.6–2.8)
Bernatsky et al.	2013	Multinational	SLE	16,409 (90)	1.19 (0.65–2.00)
Bjørneklett et al.	2007	Norway	Membranous nephropathy	161 (36)	2.74 (0.07–15.3)
Brinton et al.	1989	USA	Perniciosus anemia	5,161 (0)	**3.21 (2.2–4.6)**
Brito-Zerón et al.	2017	Spain	Sjögren’s syndrome	1,239 (92)	2.23 (0.93–5.36)
Chang et al.	2014	South Korea	RA	2,104 (82)	0.663 (0.327–0.998)
Chang et al.	2015	South Korea	SLE	1,052 (89)	0.597 (0.123–1.744)
Chang et al.	2016	South Korea	SSc	274 (88)	0.898 (0.109–3.245)
Chang et al.	2017	South Korea	Dermatomyositis	107 (81)	1.629 (0.041–9.076)
Chang et al.	2018	South Korea	Polymyositis	49 (40)	2.113 (0.054–11.774)
Chen et al.	2010	Taiwan	SLE	11,763 (88)	**2.08 (1.97–2.19)**
Collin et al.	1996	Finland	Celiac disease	383 (73)	0 (0–6.18)
			Dermatitis herpetiformis	305 (47)	2.86 (0.35–10.3)
Dreyer et al.	2011	Denmark	SLE	576 (88)	N/A
Goldrace et al.	2007	UK	Celiac disease	1,997 (NA)	1.83 (0.79–3.62)
			Crohn’s disease	5,127 (NA)	0.96 (0.44–1.83)
			Ulcerative colitis	6,990 (NA)	0.78 (0.39–1.41)
Gridley et al.	1993	Sweden	RA	11,683 (68)	0.63 (0.5–0.9)
Harding et al.	2015	Australia	T1DM	80,676 (48)	**1.37 (1.01–1.87)**
Hashimoto et al.	2012	Japan	SSc	405 (93)	0.84 −0.11–1.79)
Hashimoto et al.	2015	Japan	RA	NA (82)	0.83 (0.65–1.02)
Hemminki et al.	2011	Sweden	Addison’s disease	1,594 (NA)	**2.74 (1.24–5.23)**
			ALS	4,262 (NA)	0.96 (0.25–2.49)
			Ankylosing spondylitis	5,173 (NA)	0.92 (0.49–1.57)
			Behcet disease	2,860 (NA)	1.66 (0.83–2.99)
			Celiac disease	4,124 (NA)	N/A
			Chronic rheumatic heart disease	16,770 (NA)	**1.4 (1.07–1.81)**
			Crohn’s disease	28,349 (NA)	0.87 (0.63–1.17)
			Graves’/hyperthyroidism	36,240 (NA)	**1.31 (1.07**–**1.59)**
			Hashimoto/hypothroidism	10,682 (NA)	1.34 (0.87–1.96)
			ITP	1,709 (NA)	**3.04 (1.09–6.66)**
			Localized scleroderma	3,128 (NA)	1.56 (0.7–2.55)
			Multiple sclerosis	12,553 (NA)	0.55 (0.28–0.97)
			Myasthenia gravis	17,974 (NA)	**1.38 (1.14–1.65)**
			PBC	835 (NA)	1.29 (0.12–4.75)
			Pernicious anemia	11,839 (NA)	**4.09 (3.36–4.94)**
			Polyarteritis nodosa	12,046 (NA)	1.02 (0.71–1.42)
			Polymyalgia rheumatica	14,745 (NA)	**1.45 (1.11–1.85)**
			Polymyositis/dermatomyositis	1,256 (NA)	2.74 (0.99–6.01)
			Psoriasis	15,592 (NA)	1.28 (0.94–1.69)
			RA	26,937 (NA)	1.07 (0.82–1.38)
			Rheumatic fever	3,458 (NA)	1.5 (0.86–2.44)
			Sarcoidosis	9,053 (NA)	1.45 (0.98–2.06)
			Sjögren’s syndrome	3,769 (NA)	1.42 (0.73–2.48)
			SLE	5,318 (NA)	1.2 (0.57–2.21)
			SSc	1,195 (NA)	1.32 (0.12–4.87)
			T1DM	20,554 (NA)	2.64 (0.83–6.21)
			Ulcerative colitis	16,363 (NA)	0.88 (0.49–1.45)
			Wegener granulomatosis	945 (NA)	0.45 (0–2.59)
Hill et al.	2001	Sweden, Denmark, Finland	Dermatomyositis	618 (NA)	**3.5 (1.7–7.3)**
			Polymyositis	914 (NA)	0.3 (0.04–1.9)
Hirano et al.	2004	Japan	IgG4-RD, AIP	113 (20)	0.75 (0.086–2.59)
Hsing et al.	1993	Sweden	Pernicious anemia	4,517 (55)	**M: 2.8 (2–3.6)** **F: 3.1 (2.3–4.1)**
Hsu et al.	2015	Taiwan	T1DM	14,619 (53)	M: 1.08 (0.63–1.72) F: 1.33 (0.73–2.24)
Ilus et al.	2014	Finland	Celiac disease	32,439 (65)	0.9 (0.63–1.23)
Isomäki et al.	1978	Finland	Ankylosing spondylitis, Rheumatoid arthritis	46,101 (75)	N/A
Ji et al.	2010	Sweden	Addison’s disease	NA	1.48 (0.47–3.48)
			ALS	NA	1.18 (0.56–2.18)
			Ankylosing spondylitis	NA	1.31 (0.85–1.92)
			Celiac disease	NA	1.2 (0.78–1.75)
			Chronic rheumatic heart disease	NA	0.52 (0.16–1.22)
			Crohn’s disease	NA	**1.41 (1.12–1.75)**
			Discoid lupus erythematosus	NA	1.75 (0.83–3.23)
			Graves’/hyperthyroidism	NA	**1.33 (1.09–1.61)**
			Hashimoto/hypothyroidism	NA	0.9 (0.61–1.27)
			Localized scleroderma	NA	1.13 (0.48–2.24)
			Multiple sclerosis	NA	1.23 (0.87–1.7)
			Myasthenia gravis	NA	**1.64 (1.07–2.41)**
			PBC	NA	0.92 (0.29–2.16)
			Pernicious anemia	NA	2.11 (0.84–4.38)
			Polymyalgia rheumatica	NA	1.32 (0.99–1.73)
			Psoriasis	NA	**1.17 (1–1.35)**
			RA	NA	**1.2 (1.02–1.41)**
			Rheumatic fever	NA	1.78 (0.81–3.39)
			Sarcoidosis	NA	**1.53 (1.09–2.07)**
			Sjögren’s syndrome	NA	0.75 (0.43–1.2)
			SLE	NA	1.08 (0.59–1.81)
			SSc	NA	1.09 (0.5–2.09)
			T1DM	NA	**1.27 (1.08–1.48)**
			Ulcerative colitis	NA	**1.39 (1.14–1.69)**
Ji et al.	2018	Sweden	Giant cell arteritis, polymyalgia rheumatica	35,918 (NA)	**1.27 (1.07–1.5)**
Kang et al.	2009	South Korea	SSc	112 (74)	**3 (1.9–4.1)**
Kirkegárd et al.	2018	Denmark	Hyperthyroidism	92,783 (83)	**1.24 (1.08–1.42)**
			Hypothyroidism	71,189 (84)	**1.49 (1.26–1.75)**
Koskinen et al.	2021	Finland	Celiac disease	1,460 (63)	1.91 (0.95–3.41)
Lee H et al.	2019	South Korea	RA	1,885 (84)	M: 1.17 (0.22–2.88) F: 2.03 (0.97–3.48)
Lim et al.	2019	Singapore	RA	1,117 (84)	1.43 (0.6–3.44)
Lööf et al.	1994	Sweden	PBC	559 (88)	1.3 (0–7.2)
Nam et al.	2019	South Korea	Ankylosing spondylitis	21,780 (0)	0.93 (0.65–1.21)
Park et al.	2014	South Korea	Takayasu arteritis	180 (87)	1.4 (0–7.9)
Shiokawa et al.	2013	Japan	AIP	108 (26)	**2.7 (1.4–3.9)**
Shu et al.	2010	Sweden	T1DM	24,052 (47)	**3.,31 (1.41–6.56)**
Silano et al.	2007	Italy	Celiac disease	3,463 (43)	**3 (1.3–4.9)**
Stockton et al.	2000	Scotland	Dermatomyositis	286 (66)	**10 (2.1–29.2)**
Swerdlow et al.	2005	UK	T1DM	29,701 (44)	1.2 (0.48–2.47)
Swerdlow et al.	2006	UK	T1DM	29,701 (44)	0.77 (0.4–1.35)
Tallbacka et al.	2018	Finland	SLE	205 (89)	1.2 (0.03–6.7)
Thomas et al.	2000	Scotland	RA	26,623 (73)	M: 1.05 (0.74–1.46) F: 0.7 (0.5–0.95)
Van Daalen et al.	2017	The Netherlands	ANCA vasculitis	203 (35)	2.37 (0.06–13.2)
Viljaama et al.	2005	Finland	Celiac disease	781 (68)	1.2 (0.2–4.5)
			Dermatitis herpetiformis	366 (48)	2.1 (0.4–6.3)
Weng et al.	2015	Taiwan	Sjögren’s syndrome	7,852 (88)	1.56 (0.75–2.86)
Yamada et al.	2011	Japan	RA	7,566 (82)	1.19 (0.8–1.7)
Yoo et al.	2018	South Korea	ANCA vasculitis	150 (69)	0.36 (0.009–2.012)
Yu et al.	2016	Taiwan	Behçet disease	1,620 (57)	N/A
			Dermatomyositis	1,119 (67)	1.88 (0.47–7.52)
			Inflammatory bowel disease	2,853 (37)	0.53 (0.13–2.11)
			Kawasaki disease	3,469 (60)	N/A
			Other vasculitis	644 (36)	N/A
			Polymyositis	811 (67)	N/A
			RA	35,182 (77)	0.92 (0.72–1.16)
			Sjögren’s syndrome	11,988 (89)	1 (0.63–1.58)
			SLE	15,623 (88)	1.88 (1.21–2.91)
			SSc	1,814 (75)	0.7 (0.17–2.79)

SIR, standardized incidence rate; AIP, autoimmune pancreatitis; IgG4-RD, immunglobulin G4-related disease; SLE, systemic lupus erythematosus; NA, not available; RA, rheumatoid arthritis; SSc, systemic sclerosis; T1DM, type 1 diabetes mellitus; ALS, amyotrophic lateral sclerosis; ITP, immune thrombocytopenic purpura; PBC, primary biliary cirrhosis; M, males; F, females; ANCA, antineutrophil cytoplasmic antibody.

Number in bold indicate statistically significant results.

### Analytical Results of Associations of Autoimmune Diseases and Gastric Cancer

**Figure 2:**
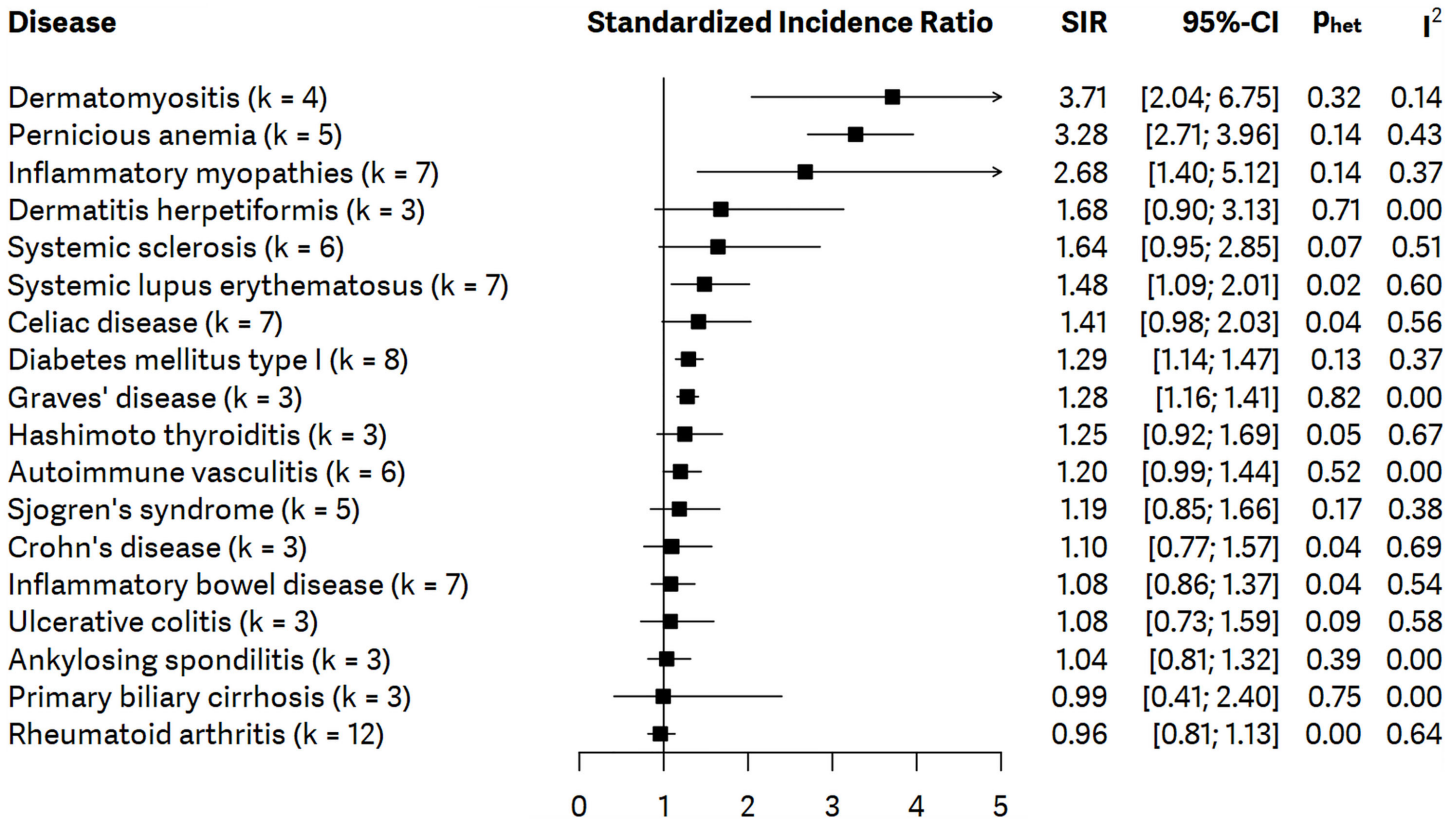
Summarizing forest plot with pooled standardized incidence ratios (SIRs), representing the incidence of gastric cancer in all patients with autoimmune disorders included in meta-analysis; *number of studies – k*.

Significantly increased incidence of gastric cancer was observed in the cases dermatomyositis (SIR = 3.71; 95% CI: 2.04, 6.75; *p* < 0.0001) based on four studies, pernicious anemia (SIR = 3.28; 95% CI: 2.71, 3.96; *p* < 0.0001) based on five studies, inflammatory myopathies (SIR = 2.68; 95% CI:1.40; 5.12; *p* = 0.0029) based on seven articles, systemic lupus erythematosus (SIR = 1.48; 95% CI: 1.09, 2.01; *p* = 0.0116) according to the analysis of seven records, diabetes mellitus type I (SIR = 1.29; 95% CI:1.14, 1.47; *p* < 0.0001) according to eight studies, and Graves’ disease (SIR = 1.28; 95% CI: 1.16, 1.41; *p* < 0.0001) in the analysis of three studies. No significant differences could be found regarding autoimmune vasculitis, celiac disease, systemic sclerosis, dermatitis herpetiformis, Hashimoto thyroiditis, Sjogren’s syndrome, inflammatory bowel disease, Crohn’s disease, rheumatoid arthritis, ulcerative colitis, ankylosing spondylitis, and primary biliary cirrhosis. Detailed results are presented in **Figure 2**.

### Subgroup Analysis Based on Gender

Diabetes mellitus type I increased the incidence of gastric cancer in female patients (SIR = 1.62; 95% CI: 1.20, 2.18) but not in male patients. Rheumatoid arthritis did not increase the incidence of gastric cancer in male or female patients. Subgroup analysis could not be performed regarding other autoimmune diseases. Results of the subgroup analysis based on gender are presented in **Supplementary Figures S39, S40**.

### Subgroup Analysis Based on the Incidence of Gastric Cancer

Pernicious anemia (SIR = 3.28; 95% CI: 2.71, 3.96), diabetes mellitus type I (SIR = 1.41; 95% CI: 1.02, 1.95), Graves’ disease (SIR = 1.28; 95% CI: 1.61, 1.41), and autoimmune vasculitis (SIR = 1.21; 95% CI: 1.01, 1.44) were associated with gastric cancer in low-incidence countries.

Systemic lupus erythematosus (SIR = 1.69; 95% CI: 1.21, 2.36) was associated with increased incidence of gastric cancer in high-incidence countries. However, in the case of dermatomyositis, subgroup analysis could not be performed, it was also associated with gastric cancer (SIR = 5.10; 95% CI: 1.90, 13.67) in low-incidence countries, based on two studies. We did not find significant statistical difference concerning the other autoimmune diseases. The detailed results of the subgroup analysis are presented in **Supplementary Figures S41–S56**.

### Qualitative Synthesis

**Figure 3:**
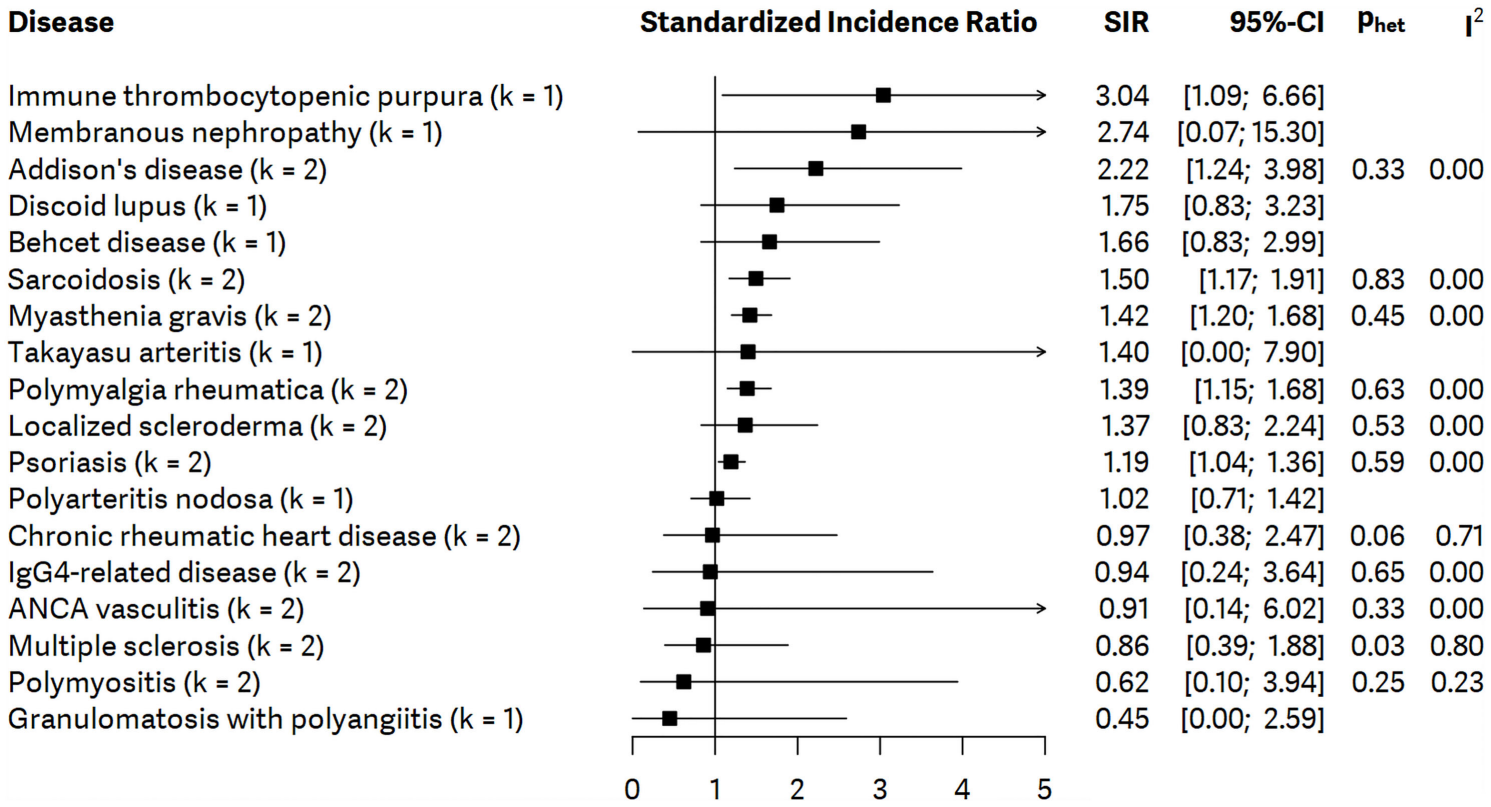
Summarizing forest plot with pooled standardized incidence ratios (SIRs), representing the incidence of gastric cancer in all patients with autoimmune disorders included in qualitative synthesis; *number of studies – k*.

Eighteen other autoimmune disorders were included in the qualitative synthesis. The individual articles found an increased incidence of gastric cancer in the cases of immune thrombocytopenic purpura (20), membranous nephropathy (21), Addison’s disease (20, 22), discoid lupus (22), Bechet’s disease (20, 23), sarcoidosis (20, 22), myasthenia gravis (20, 22), Takayasu arteritis (24), polymyalgia rheumatica (20, 22), localized scleroderma (20, 22), and psoriasis (20, 22). Chronic rheumatic heart disease (20, 22), IgG4-related disease (25, 26), ANCA-vasculitis (27, 28), multiple sclerosis (20, 22), and granulomatosis with polyangiitis (20) seem not to be associated with elevated incidence of gastric cancer. The detailed results of the qualitative synthesis are presented in **Figure 3**.

### Risk of Bias Assessment

Results and a detailed description of the risk of bias assessment according to the QUIPS tool are presented in **Supplementary Table S2**.

Publication bias was assessed for rheumatoid arthritis by the Egger’s test, which does not indicate the presence of Funnel plot asymmetry. Therefore, we concluded that no publication bias was present. Funnel plot is presented in **Supplementary Figure S57**.

### Statistical Heterogeneity

The heterogeneity analysis proved to be significant in the analysis of rheumatoid arthritis (*I* ^2^ = 0.64; *p* = 0.00), inflammatory bowel disease (*I* ^2^ = 0.54; *p* = 0.04), systemic lupus erythematosus (*I* ^2^ = 0.60; *p* = 0.02), coeliac disease (*I* ^2^ = 0.56; *p* = 0.04), Crohn’s disease (*I* ^2^ = 0.69; *p* = 0.04), and rheumatoid arthritis in the case of subgroup analysis of female patients (*I* ^2^ = 0.69; *p*=0.01). Other comparisons did not prove to be significant regarding heterogeneity. Detailed results of heterogeneity are presented in **Supplementary Figure S1**.

## Discussion

This meta-analysis, including data of 499,427 patients collected from 43 studies, was conducted to understand the relationship between autoimmunity and gastric cancer. Based on our results, the incidence of gastric cancer significantly increased in patients with pernicious anemia, Graves’ disease, dermatomyositis, diabetes mellitus type I, inflammatory myopathies, and systemic lupus erythematosus.

In line with our results, the literature suggests that patients with dermatomyositis, rheumatoid arthritis, scleroderma, systemic lupus erythematosus, or diabetes mellitus type I may have an increased risk for developing multiple cancers (29–34). Positive associations have been observed between various gastrointestinal tumors and rheumatoid arthritis, systemic lupus erythematosus, Sjögren’s syndrome, celiac disease, idiopathic inflammatory myositis, and systemic sclerosis (35–39).

A recent meta-analysis described a correlation between autoimmune disorders and increased risk of gastric cancer (40). Song et al. concluded that patients with dermatomyositis, pernicious anemia, Addison’s disease, dermatitis herpetiformis, IgG4-related disease, primary biliary cirrhosis, diabetes mellitus type I, systemic lupus erythematosus, and Graves’ disease had elevated risk for developing gastric neoplasms.

Pernicious anemia has been demonstrated as a risk factor for gastric cancer (41) since it correlates with autoimmune gastritis and results from gastric mucosal damage. This pathomechanism has been modeled in mice and has suggested an association between autoimmunity and carcinogenesis (14). Autoimmune thyroiditis, diabetes mellitus type I, vitiligo, and Addison disease are frequently associated with pernicious anemia.

An increase in the incidence of autoimmune diseases has been observed recently parallelly with the increasing incidence of cancers. The autoimmune inflammation often correlates with the tumorous disorder of the affected organ. This phenomenon is most conspicuous in people below 50 years of age, and it affects females more considering the development of gastric cancer (7, 8).

Although autoimmune processes can play a significant role in developing different cancers, the exact pathomechanisms remain unclear. Several common factors can be identified, such as immunosuppression/dysregulation, infections, dietary habits, environmental factor, and chronic inflammation. These factors can induce chronic cell damage and can trigger either autoimmune conditions or cancer (12). Autoimmune disorders may lead to antigen specificity-driven tissue damage causing chronic inflammation, whose role in carcinogenesis is well known and precedes the tumor formation in time (42).

Regarding the strengths of our meta-analysis, we included a large number of cohort studies. Many of our analytical results proved to be significant. This comprehensive work contains wide coverage of AI disorders from 15 different countries and four continents of the currently available literature so far. Following the PRISMA Statement and a rigorous methodology, the quality is secured. The key questions of this study were not widely investigated recently; thus, most of our findings are novel.

The formerly mentioned meta-analysis (40) discussing the question of interest had several limitations. Compared with that work, a more general search key was used in our study, which allowed us to find a higher number of relevant records. Our search was conducted in four databases compared with the two in the previous work, which also contributed to the identification of further eligible studies. They calculate pooled relative risk ratios (RR) with 95% CI; however, hazard ratios, SIRs, RRs, and standardized mortality rates were pooled into RR. Statistical analysis of our study is also more coherent as only SIR-s were calculated consistently (43, 44).

However, our analysis has some limitations, which should be considered for a correct interpretation. Firstly, other risk factors for gastric cancer, such as *H. pylori* infection status, smoking, dietary habits, obesity, occupational exposure to dust, high-temperature particulates, and metals such as chromium VI, gastric surgery (by-pass), and Epstein-Barr infection could be present that were not measured or reported. We also did not have information about drugs taken for autoimmune disorders, so how it may affect the outcome is unknown. However, according to 10 included articles, the mean time interval from the diagnosis of AI disorder to the diagnosis of cancer is 2–7.4 years. Although, the mentioned time intervals refer to the development of any type of cancer in general, not only to gastric cancer.

The diagnosis of AI diseases in countries could be different, which could create significant heterogeneity in some of the analyses. The presence of ascertainment cannot be ruled out, since people with an autoimmune disease are subjected to medical examinations more frequently, than the general population, which may lead to a greater number of cancer diagnoses. The low number of enrolled studies regarding certain autoimmune disorders, which could not be meta-analyzed, is also a further limitation. The risk of bias assessment deemed in case of multiple domains as not low overall risk of bias too.

Subgroup analyses regarding the type of gastric cancer could not be performed, because there were no details available on histological type, or location of cancer. However, Ji et al. described that a few autoimmune diseases is an important risk factor for gastric cancer, mainly for corpus cancer (22). Sensitivity analysis was carried out to define the strength of confounding factors, such as *H. pylori* infection, which results suggest the examined association is unlikely to be solely because of confounding.

Most of the included studies originate from either North Europe (where incidence of autoimmunity could be higher compared with other populations) or Asia (where *H. pylori* infection and/or gastric cancer could be more prevalent). To address this problem, we performed subgroup analyses based on low- or high-incidence countries of gastric cancer. The results of the subgroup analysis reassert our main results, namely pernicious anemia, diabetes mellitus type I, Graves’ disease, and autoimmune vasculitis were associated with gastric cancer in low-incidence countries.

## Conclusion

Our meta-analysis of 39 articles concludes that pernicious anemia, Graves’ disease, dermatomyositis, diabetes mellitus type I, inflammatory myopathies, and systemic lupus erythematosus are associated with higher incidence rates of gastric cancer. For clinical practice, close gastroenterological follow-up or routinely performed gastroscopy and application of other diagnostic measures may be cost-effective and clinically helpful for patients diagnosed with these six autoimmune diseases. Based on the importance of the problem, conducting further clinical trials on this topic is essential.

## Data Availability Statement

The original contributions presented in the study are included in the article/**Supplementary Material**. Further inquiries can be directed to the corresponding author.

## Author Contributions

NZ: conceptualization, project administration, formal analysis, and writing—original draft. EO: conceptualization, methodology, and statistical analysis. PH: conceptualization and writing—review and editing. SV: conceptualization, data curation, and writing—review and editing. NV: conceptualization, data curation, and writing—review and editing. SK: conceptualization, methodology, and writing—review and editing. LF: conceptualization, visualization, and writing—review and editing. LS: conceptualization, methodology, visualization, and writing—original draft. JC: conceptualization, supervision, and writing—original draft. All authors have participated sufficiently to take public responsibility for the content, including participation in the concept, design, analysis, writing, or revision of the manuscript. All authors contributed to the article and approved the submitted version.

## Conflict of Interest

The authors declare that the research was conducted in the absence of any commercial or financial relationships that could be construed as a potential conflict of interest.

## Publisher’s Note

All claims expressed in this article are solely those of the authors and do not necessarily represent those of their affiliated organizations, or those of the publisher, the editors and the reviewers. Any product that may be evaluated in this article, or claim that may be made by its manufacturer, is not guaranteed or endorsed by the publisher.
