# Supplementary material for: Six Autoimmune Disorders Are Associated With Increased Incidence of Gastric Cancer: A Systematic Review and Meta-Analysis of Half a Million Patients
Source: Front Immunol. 2021 Nov 23;12:750533. doi: 10.3389/fimmu.2021.750533 (PMC8650004; doi:10.3389/fimmu.2021.750533)
Supplement: Supplementary file 1 [file DataSheet_1.docx]

Supplementary Material

Six autoimmune disorders are associated with increased incidence of gastric cancer: A systematic review and meta-analysis of half a million patients.

**Noémi Zádori^1,2^, Lajos Szakó^1,2^, Szilárd Váncsa^1,2,5^, Nóra Vörhendi^1,2^, Eduard Oštarijaš^1^, Szabolcs Kiss^1,3,4^, Levente Frim^1^,  Péter Hegyi^1,2,5,6^ and József Czimmer^1,7^**

^1.^ Institute for Translational Medicine, Medical School, University of Pécs, Pécs, Hungary; info@tm-centre.org

^2.^ János Szentágothai Research Centre, University of Pécs, Pécs, Hungary; info@szkk.pte.hu

^3.^ Doctoral School of Clinical Medicine, University of Szeged, Szeged, Hungary

^4.^ Heim Pál National Pediatric Institute, Budapest, Hungary

^5^. Centre for Translational Medicine, Semmelweis University, Budapest, Hungary

^6.^ Division of Pancreatic Diseases, Heart and Vascular Center, Semmelweis University, Budapest, Hungary

^7.^ Division of Gastroenterology, First Department of Medicine, Medical School, University of Pécs, Pécs, Hungary; [info@pte.hu](mailto:info@pte.hu)

**Table of content**

**Supplementary Table 1.** PRISMA checklist

**Supplementary Table 2.** Risk of bias assessment using QUIPS tools

**Supplementary Table 3.** Detailed risk of bias assessment of prognostic factors

**Supplementary Figure 1.** The standardized incidence ratios of gastric cancer in all examined autoimmune disorder – summarizing plot

**Supplementary Figure 2-19.** Forest plots about the standardized incidence ratios of gastric cancer in those autoimmune disorders which were included in meta-analysis

**Supplementary Figure 20-38.** Forest plots about the standardized incidence ratios of gastric cancer in those autoimmune disorders which were included in systematic review

**Supplementary Figure 39-40.** Forest plots about subgroup analyses based on gender

**Supplementary Figure 41-56.** Forest plots about subgroup analyses based on high incidence or low incidence countries of gastric cancer.

**Supplementary Figure 57.** Funnel plot for rheumatoid arthritis.

**Supplementary References**

**Supplementary Table 1.** Prisma checklist ^1^

| **Section and Topic** | **Item #** | **Checklist item** | **Location where item is reported** |
| --- | --- | --- | --- |
| **TITLE** | | |  |
| Title | 1 | Identify the report as a systematic review. | 1 |
| **ABSTRACT** | | |  |
| Abstract | 2 | See the PRISMA 2020 for Abstracts checklist. | 2, suppl. |
| **INTRODUCTION** | | |  |
| Rationale | 3 | Describe the rationale for the review in the context of existing knowledge. | 3 |
| Objectives | 4 | Provide an explicit statement of the objective(s) or question(s) the review addresses. | 3 |
| **METHODS** | | |  |
| Eligibility criteria | 5 | Specify the inclusion and exclusion criteria for the review and how studies were grouped for the syntheses. | 4 |
| Information sources | 6 | Specify all databases, registers, websites, organisations, reference lists and other sources searched or consulted to identify studies. Specify the date when each source was last searched or consulted. | 4 |
| Search strategy | 7 | Present the full search strategies for all databases, registers and websites, including any filters and limits used. | 4 |
| Selection process | 8 | Specify the methods used to decide whether a study met the inclusion criteria of the review, including how many reviewers screened each record and each report retrieved, whether they worked independently, and if applicable, details of automation tools used in the process. | 4 |
| Data collection process | 9 | Specify the methods used to collect data from reports, including how many reviewers collected data from each report, whether they worked independently, any processes for obtaining or confirming data from study investigators, and if applicable, details of automation tools used in the process. | 5 |
| Data items | 10a | List and define all outcomes for which data were sought. Specify whether all results that were compatible with each outcome domain in each study were sought (e.g. for all measures, time points, analyses), and if not, the methods used to decide which results to collect. | 5 |
|  | 10b | List and define all other variables for which data were sought (e.g. participant and intervention characteristics, funding sources). Describe any assumptions made about any missing or unclear information. | 5 |
| Study risk of bias assessment | 11 | Specify the methods used to assess risk of bias in the included studies, including details of the tool(s) used, how many reviewers assessed each study and whether they worked independently, and if applicable, details of automation tools used in the process. | 5, suppl. |
| Effect measures | 12 | Specify for each outcome the effect measure(s) (e.g. risk ratio, mean difference) used in the synthesis or presentation of results. | 5 |
| Synthesis methods | 13a | Describe the processes used to decide which studies were eligible for each synthesis (e.g. tabulating the study intervention characteristics and comparing against the planned groups for each synthesis (item #5)). | 5 |
|  | 13b | Describe any methods required to prepare the data for presentation or synthesis, such as handling of missing summary statistics, or data conversions. | 5 |
|  | 13c | Describe any methods used to tabulate or visually display results of individual studies and syntheses. | 5 |
|  | 13d | Describe any methods used to synthesize results and provide a rationale for the choice(s). If meta-analysis was performed, describe the model(s), method(s) to identify the presence and extent of statistical heterogeneity, and software package(s) used. | 5 |
|  | 13e | Describe any methods used to explore possible causes of heterogeneity among study results (e.g. subgroup analysis, meta-regression). | 5 |
|  | 13f | Describe any sensitivity analyses conducted to assess robustness of the synthesized results. | - |
| Reporting bias assessment | 14 | Describe any methods used to assess risk of bias due to missing results in a synthesis (arising from reporting biases). | 5 |
| Certainty assessment | 15 | Describe any methods used to assess certainty (or confidence) in the body of evidence for an outcome. | 5 |
| **RESULTS** | | |  |
| Study selection | 16a | Describe the results of the search and selection process, from the number of records identified in the search to the number of studies included in the review, ideally using a flow diagram. | 6, figure 1. |
|  | 16b | Cite studies that might appear to meet the inclusion criteria, but which were excluded, and explain why they were excluded. | figure 1 |
| Study characteristics | 17 | Cite each included study and present its characteristics. | table 1, suppl |
| Risk of bias in studies | 18 | Present assessments of risk of bias for each included study. | 7, suppl. |
| Results of individual studies | 19 | For all outcomes, present, for each study: (a) summary statistics for each group (where appropriate) and (b) an effect estimate and its precision (e.g. confidence/credible interval), ideally using structured tables or plots. | 7, suppl. |
| Results of syntheses | 20a | For each synthesis, briefly summarise the characteristics and risk of bias among contributing studies. | 7, suppl. |
|  | 20b | Present results of all statistical syntheses conducted. If meta-analysis was done, present for each the summary estimate and its precision (e.g. confidence/credible interval) and measures of statistical heterogeneity. If comparing groups, describe the direction of the effect. | 6 |
|  | 20c | Present results of all investigations of possible causes of heterogeneity among study results. | 7 |
|  | 20d | Present results of all sensitivity analyses conducted to assess the robustness of the synthesized results. | - |
| Reporting biases | 21 | Present assessments of risk of bias due to missing results (arising from reporting biases) for each synthesis assessed. | 7, suppl |
| Certainty of evidence | 22 | Present assessments of certainty (or confidence) in the body of evidence for each outcome assessed. | 6 |
| **DISCUSSION** | | |  |
| Discussion | 23a | Provide a general interpretation of the results in the context of other evidence. | 8-9 |
|  | 23b | Discuss any limitations of the evidence included in the review. | 9 |
|  | 23c | Discuss any limitations of the review processes used. | 9 |
|  | 23d | Discuss implications of the results for practice, policy, and future research. | 9 |
| **OTHER INFORMATION** | | |  |
| Registration and protocol | 24a | Provide registration information for the review, including register name and registration number, or state that the review was not registered. | 4 |
|  | 24b | Indicate where the review protocol can be accessed, or state that a protocol was not prepared. | 4 |
|  | 24c | Describe and explain any amendments to information provided at registration or in the protocol. | 4 |
| Support | 25 | Describe sources of financial or non-financial support for the review, and the role of the funders or sponsors in the review. | 10 |
| Competing interests | 26 | Declare any competing interests of review authors. | 10 |
| Availability of data, code and other materials | 27 | Report which of the following are publicly available and where they can be found: template data collection forms; data extracted from included studies; data used for all analyses; analytic code; any other materials used in the review. | - |

*From:*  Page MJ, McKenzie JE, Bossuyt PM, Boutron I, Hoffmann TC, Mulrow CD, et al. The PRISMA 2020 statement: an updated guideline for reporting systematic reviews. BMJ 2021;372:n71. doi: 10.1136/bmj.n71

**Supplementary Table 2. Risk of bias assessment using the QUIPS tool ^2^**

| **Authors** | **1** | **2** | **3** | **4** | **5** | **6** | **Overall** | **Included in meta-analysis** |
| --- | --- | --- | --- | --- | --- | --- | --- | --- |
| Asano et al. ^3^ |  |  |  |  |  |  |  | no |
| Askling et al. ^4^ |  |  |  |  |  |  |  | yes |
| Bernatsky et al. ^5^ |  |  |  |  |  |  |  | yes |
| Bjorneklett et al. ^6^ |  |  |  |  |  |  |  | no |
| Brinton et al. ^7^ |  |  |  |  |  |  |  | yes |
| Briton-Zeron et al. ^8^ |  |  |  |  |  |  |  | yes |
| Chang SH et al. ^9^ |  | N/A |  |  |  |  |  | yes |
| Chen JY et al. ^10^ |  |  |  |  |  |  |  | yes |
| Collin et al. ^11^ |  |  |  |  |  |  |  | yes |
| Dreyer et al. ^12^ |  |  |  |  |  |  |  | yes |
| Goldrace et al. ^13^ |  |  |  |  |  |  |  | yes |
| Gridley et al. ^14^ |  |  |  |  |  |  |  | yes |
| Harding et al. ^15^ |  |  |  |  |  |  |  | yes |
| Hashimoto et al.-1 ^16^ |  |  |  |  |  |  |  | yes |
| Hashimoto et al.-2 ^17^ |  |  |  |  |  |  |  | yes |
| Hemminki et al. ^18^ |  |  |  |  |  |  |  | yes |
| Hill et al. ^19^ |  |  |  |  |  |  |  | yes |
| Hirano et al. ^20^ |  |  |  |  |  |  |  | no |
| Hsing et al. ^21^ |  |  |  |  |  |  |  | yes |
| Hsu et al. ^22^ |  |  |  |  |  |  |  | yes |
| Ilus et al. ^23^ |  |  |  |  |  |  |  | yes |
| Ji et al. ^24^ |  |  |  |  |  |  |  | yes |
| KH Yu et al. ^25^ |  |  |  |  |  |  |  | yes |
| Kang et al. ^26^ |  |  |  |  |  |  |  | yes |
| Kirkegaard et al. ^27^ |  |  |  |  |  |  |  | yes |
| Koskinen et al. ^28^ |  | N/A |  |  |  |  |  | yes |
| Lee H et al. ^29^ |  |  |  |  |  |  |  | yes |
| Lööf et al. ^30^ |  |  |  |  |  |  |  | yes |
| Nam et al. ^31^ |  |  |  |  |  |  |  | yes |
| Park et al. ^32^ |  |  |  |  |  |  |  | yes |
| Shiokawa et al. ^33^ |  | N/A |  |  |  |  |  | no |
| Shu X et al. ^34^ |  |  |  |  |  |  |  | yes |
| Silano et al. ^35^ |  |  |  |  |  |  |  | yes |
| Stockton et al. ^36^ |  |  |  |  |  |  |  | yes |
| Swerdlow et al. ^37^ |  |  |  |  |  |  |  | yes |
| Tallbacka et al. ^38^ |  |  |  |  |  |  |  | yes |
| Thomas et al. ^39^ |  |  |  |  |  |  |  | yes |
| Van Daalen et al. ^40^ |  |  |  |  |  |  |  | yes |
| Viljaama et al. ^41^ |  |  |  |  |  |  |  | yes |
| Weng et al. ^42^ |  |  |  |  |  |  |  | yes |
| Xin Long Lim et al. ^43^ |  |  |  |  |  |  |  | yes |
| Yamada et al. ^44^ |  |  |  |  |  |  |  | yes |
| Yoo et al. ^45^ |  | **N/A** |  |  |  |  |  | yes |

*1.study population, 2. Study attrition, 3. Prognostic factor measurement, 4. Outcome measurement, 5. Study confounding, 6. Statistical analysis and reporting*

Results of QUIPS score.

| Low risk of bias |
| --- |
| High risk of bias |
| Moderate risk of bias |

Study participation measurement: Low risk of bias was given if a clear description of basic characteristics of study participants was reported, including inclusion and exclusion criteria. If only a subpopulation was included (e.g. based on sex) high risk of bias was declared. If the work did not provide a description, unclear risk of bias was attributed.

Prognostic factor measurement: Low risk of bias was given if a clear definition of the autoimmune disorder was provided. In the case of unclear risk of bias no information about the definition of the autoimmune disorder was available. Studies, which described a definition not according to the international definitions of autoimmune diseases were defined as articles of high risk.

Outcome measurement: Low risk of bias was given if a clear definition (histological type of cancer, stage, diagnostic approach), according to the accepted guidelines was provided. In the case of unclear risk of bias no information about the definition of the outcome was available. Studies, which described a definition not according to the accepted definitions of outcomes were defined as high risk carrying articles.

Study confounding measurement: Low risk of bias was given, because the definition of SIR includes age and gender, which are considered to be as confounders.

Overall risk of bias: Overall the risk of a study was deemed to be low if every domain was low. In the case of at least one high risk of bias domain, the overall risk was declared to be high. In every other case, an overall unclear risk of bias was given.

**Supplementary Table 3.** Detailed risk of bias assessment of prognostic factors

| **Authors** | **Prognostic factor measurement** | | | | | | | | | | | | | | | | | | | | | | | | | | | | | | | | |
| --- | --- | --- | --- | --- | --- | --- | --- | --- | --- | --- | --- | --- | --- | --- | --- | --- | --- | --- | --- | --- | --- | --- | --- | --- | --- | --- | --- | --- | --- | --- | --- | --- | --- |
|  | **T1DM** | **PA** | **CD** | **DM** | **PM** | **SLE** | **RA** | **SJ** | **AS** | **Ssc** | **DH** | **CSD** | **AIV** | **HT** | **GD** | **IBD** | **IM** | **PBC** | **UC** | **AIP** | **MN** | **AD** | **BD** | **CRHD** | **ITP** | **LS** | **MS** | **MG** | **PR** | **PS** | **SR** | **IgG4RD** | **DL** |
|  |  |  |  |  |  |  |  |  |  |  |  |  |  |  |  |  |  |  |  |  |  |  |  |  |  |  |  |  |  |  |  |  |  |
| Asano et al. | n.a. | n.a. | n.a. | n.a. | n.a. | n.a. | n.a. | n.a. | n.a. | n.a. | n.a. | n.a. | n.a. | n.a. | n.a. | n.a. | n.a. | n.a. | n.a. | n.a. | n.a. | n.a. | n.a. | n.a. | n.a. | n.a. | n.a. | n.a. | n.a. | n.a. | n.a. |  | n.a. |
| Askling et al. | n.a. | n.a. |  | n.a. | n.a. | n.a. | n.a. | n.a. | n.a. | n.a. |  | n.a. | n.a. | n.a. | n.a. | n.a. | n.a. | n.a. | n.a. | n.a. | n.a. | n.a. | n.a. | n.a. | n.a. | n.a. | n.a. | n.a. | n.a. | n.a. | n.a. | n.a. | n.a. |
| Bernatsky et al. | n.a. | n.a. | n.a. | n.a. | n.a. |  | n.a. | n.a. | n.a. | n.a. | n.a. | n.a. | n.a. | n.a. | n.a. | n.a. | n.a. | n.a. | n.a. | n.a. | n.a. | n.a. | n.a. | n.a. | n.a. | n.a. | n.a. | n.a. | n.a. | n.a. | n.a. | n.a. | n.a. |
| Bjorneklett et al. | n.a. | n.a. | n.a. | n.a. | n.a. | n.a. | n.a. | n.a. | n.a. | n.a. | n.a. | n.a. | n.a. | n.a. | n.a. | n.a. | n.a. | n.a. | n.a. | n.a. |  | n.a. | n.a. | n.a. | n.a. | n.a. | n.a. | n.a. | n.a. | n.a. | n.a. | n.a. | n.a. |
| Brinton et al. | n.a. |  | n.a. | n.a. | n.a. | n.a. | n.a. | n.a. | n.a. | n.a. | n.a. | n.a. | n.a. | n.a. | n.a. | n.a. | n.a. | n.a. | n.a. | n.a. | n.a. | n.a. | n.a. | n.a. | n.a. | n.a. | n.a. | n.a. | n.a. | n.a. | n.a. | n.a. | n.a. |
| Briton-Zeron et al. | n.a. | n.a. | n.a. | n.a. | n.a. | n.a. | n.a. |  | n.a. | n.a. | n.a. | n.a. | n.a. | n.a. | n.a. | n.a. | n.a. | n.a. | n.a. | n.a. | n.a. | n.a. | n.a. | n.a. | n.a. | n.a. | n.a. | n.a. | n.a. | n.a. | n.a. | n.a. | n.a. |
| Chang SH et al. | n.a. | n.a. | n.a. |  |  |  |  | n.a. | n.a. |  | n.a. | n.a. | n.a. | n.a. | n.a. | n.a. | n.a. | n.a. | n.a. | n.a. | n.a. | n.a. | n.a. | n.a. | n.a. | n.a. | n.a. | n.a. | n.a. | n.a. | n.a. | n.a. | n.a. |
| Chen JY et al. | n.a. | n.a. | n.a. | n.a. | n.a. |  | n.a. | n.a. | n.a. | n.a. | n.a. | n.a. | n.a. | n.a. | n.a. | n.a. | n.a. | n.a. | n.a. | n.a. | n.a. | n.a. | n.a. | n.a. | n.a. | n.a. | n.a. | n.a. | n.a. | n.a. | n.a. | n.a. | n.a. |
| Collin et al. | n.a. | n.a. |  |  | n.a. | n.a. | n.a. | n.a. | n.a. | n.a. | n.a. | n.a. | n.a. | n.a. | n.a. | n.a. | n.a. | n.a. | n.a. | n.a. | n.a. | n.a. | n.a. | n.a. | n.a. | n.a. | n.a. | n.a. | n.a. | n.a. | n.a. | n.a. | n.a. |
| Dreyer et al. | n.a. | n.a. | n.a. | n.a. | n.a. |  | n.a. | n.a. | n.a. |  | n.a. | n.a. | n.a. | n.a. | n.a. | n.a. | n.a. | n.a. | n.a. | n.a. | n.a. | n.a. | n.a. | n.a. | n.a. | n.a. | n.a. | n.a. | n.a. | n.a. | n.a. | n.a. | n.a. |
| Goldrace et al. | n.a. | n.a. |  | n.a. | n.a. | n.a. | n.a. | n.a. | n.a. | n.a. | n.a. |  | n.a. | n.a. | n.a. | n.a. | n.a. | n.a. |  | n.a. | n.a. | n.a. | n.a. | n.a. | n.a. | n.a. | n.a. | n.a. | n.a. | n.a. | n.a. | n.a. | n.a. |
| Gridley et al. | n.a. | n.a. | n.a. | n.a. | n.a. | n.a. |  | n.a. | n.a. | n.a. | n.a. | n.a. | n.a. | n.a. | n.a. | n.a. | n.a. | n.a. | n.a. | n.a. | n.a. | n.a. | n.a. | n.a. | n.a. | n.a. | n.a. | n.a. | n.a. | n.a. | n.a. | n.a. | n.a. |
| Harding et al. |  | n.a. | n.a. | n.a. | n.a. | n.a. | n.a. | n.a. | n.a. | n.a. | n.a. | n.a. | n.a. | n.a. | n.a. | n.a. | n.a. | n.a. | n.a. | n.a. | n.a. | n.a. | n.a. | n.a. | n.a. | n.a. | n.a. | n.a. | n.a. | n.a. | n.a. | n.a. | n.a. |
| Hashimoto et al.-1 | n.a. | n.a. | n.a. | n.a. | n.a. | n.a. | n.a. | n.a. | n.a. |  | n.a. | n.a. | n.a. | n.a. | n.a. | n.a. | n.a. | n.a. | n.a. | n.a. | n.a. | n.a. | n.a. | n.a. | n.a. | n.a. | n.a. | n.a. | n.a. | n.a. | n.a. | n.a. | n.a. |
| Hashimoto et al.-2 | n.a. | n.a. | n.a. | n.a. | n.a. | n.a. |  | n.a. | n.a. | n.a. | n.a. | n.a. | n.a. | n.a. | n.a. | n.a. | n.a. | n.a. | n.a. | n.a. | n.a. | n.a. | n.a. | n.a. | n.a. | n.a. | n.a. | n.a. | n.a. | n.a. | n.a. | n.a. | n.a. |
| Hemminki et al. |  |  | n.a. |  |  |  |  |  | n.a. |  | n.a. | n.a. |  | n.a. | n.a. | n.a. | n.a. | n.a. | n.a. | n.a. | n.a. |  |  |  | n.a. |  |  |  |  |  |  | n.a. | n.a. |
| Hill et al. | n.a. | n.a. | n.a. |  |  | n.a. | n.a. | n.a. | n.a. | n.a. | n.a. | n.a. | n.a. | n.a. | n.a. | n.a. | n.a. | n.a. | n.a. | n.a. | n.a. | n.a. | n.a. | n.a. | n.a. | n.a. | n.a. | n.a. | n.a. | n.a. | n.a. | n.a. | n.a. |
| Hirano et al. | n.a. | n.a. | n.a. | n.a. | n.a. | n.a. | n.a. | n.a. | n.a. | n.a. | n.a. | n.a. | n.a. | n.a. | n.a. | n.a. | n.a. | n.a. | n.a. |  | n.a. | n.a. | n.a. | n.a. | n.a. | n.a. | n.a. | n.a. | n.a. | n.a. | n.a. |  | n.a. |
| Hsing et al. | n.a. |  | n.a. | n.a. | n.a. | n.a. | n.a. | n.a. | n.a. | n.a. | n.a. | n.a. | n.a. | n.a. | n.a. | n.a. | n.a. | n.a. | n.a. | n.a. | n.a. | n.a. | n.a. | n.a. | n.a. | n.a. | n.a. | n.a. | n.a. | n.a. | n.a. | n.a. | n.a. |
| Hsu et al. |  | n.a. | n.a. | n.a. | n.a. | n.a. | n.a. | n.a. | n.a. | n.a. | n.a. | n.a. | n.a. | n.a. | n.a. | n.a. | n.a. | n.a. | n.a. | n.a. | n.a. | n.a. | n.a. | n.a. | n.a. | n.a. | n.a. | n.a. | n.a. | n.a. | n.a. | n.a. | n.a. |
| Ilus et al. | n.a. | n.a. |  | n.a. | n.a. | n.a. | n.a. | n.a. | n.a. | n.a. | n.a. | n.a. | n.a. | n.a. | n.a. | n.a. | n.a. | n.a. | n.a. | n.a. | n.a. | n.a. | n.a. | n.a. | n.a. | n.a. | n.a. | n.a. | n.a. | n.a. | n.a. | n.a. | n.a. |
| Ji et al. |  |  |  | n.a. | n.a. |  |  |  | n.a. |  | n.a. |  | n.a. |  |  | n.a. | n.a. |  |  | n.a. | n.a. |  | n.a. |  | n.a. |  |  |  |  |  |  | n.a. |  |
| KH Yu et al. | n.a. | n.a. | n.a. |  | n.a. | n.a. |  |  | n.a. |  | n.a. | n.a. |  | n.a. | n.a. |  |  | n.a. | n.a. | n.a. | n.a. | n.a. |  | n.a. | n.a. | n.a. | n.a. | n.a. | n.a. | n.a. | n.a. | n.a. | n.a. |
| Kang et al. | n.a. | n.a. | n.a. | n.a. | n.a. | n.a. | n.a. | n.a. | n.a. |  | n.a. | n.a. | n.a. | n.a. | n.a. | n.a. | n.a. | n.a. | n.a. | n.a. | n.a. | n.a. | n.a. | n.a. | n.a. | n.a. | n.a. | n.a. | n.a. | n.a. | n.a. | n.a. | n.a. |
| Kirkegaard et al. | n.a. | n.a. | n.a. | n.a. | n.a. | n.a. | n.a. | n.a. | n.a. | n.a. | n.a. | n.a. | n.a. |  | n.a. | n.a. | n.a. | n.a. | n.a. | n.a. | n.a. | n.a. | n.a. | n.a. | n.a. | n.a. | n.a. | n.a. | n.a. | n.a. | n.a. | n.a. | n.a. |
| Koskinen et al. | n.a. | n.a. |  | n.a. | n.a. | n.a. | n.a. | n.a. | n.a. | n.a. | n.a. | n.a. | n.a. | n.a. | n.a. | n.a. | n.a. | n.a. | n.a. | n.a. | n.a. | n.a. | n.a. | n.a. | n.a. | n.a. | n.a. | n.a. | n.a. | n.a. | n.a. | n.a. | n.a. |
| Lee H et al. | n.a. | n.a. | n.a. | n.a. | n.a. | n.a. |  | n.a. | n.a. | n.a. | n.a. | n.a. | n.a. | n.a. | n.a. | n.a. | n.a. | n.a. | n.a. | n.a. | n.a. | n.a. | n.a. | n.a. | n.a. | n.a. | n.a. | n.a. | n.a. | n.a. | n.a. | n.a. | n.a. |
| Lööf et al. | n.a. | n.a. | n.a. | n.a. | n.a. | n.a. | n.a. | n.a. | n.a. | n.a. | n.a. | n.a. | n.a. | n.a. | n.a. | n.a. | n.a. |  | n.a. | n.a. | n.a. | n.a. | n.a. | n.a. | n.a. | n.a. | n.a. | n.a. | n.a. | n.a. | n.a. | n.a. | n.a. |
| Nam et al. | n.a. | n.a. | n.a. | n.a. | n.a. | n.a. | n.a. | n.a. |  | n.a. | n.a. | n.a. | n.a. | n.a. | n.a. | n.a. | n.a. | n.a. | n.a. | n.a. | n.a. | n.a. | n.a. | n.a. | n.a. | n.a. | n.a. | n.a. | n.a. | n.a. | n.a. | n.a. | n.a. |
| Park et al. | n.a. | n.a. | n.a. | n.a. | n.a. | n.a. | n.a. | n.a. | n.a. | n.a. | n.a. | n.a. |  | n.a. | n.a. | n.a. | n.a. | n.a. | n.a. | n.a. | n.a. | n.a. | n.a. | n.a. | n.a. | n.a. | n.a. | n.a. | n.a. | n.a. | n.a. | n.a. | n.a. |
| Shiokawa et al. | n.a. | n.a. | n.a. | n.a. | n.a. | n.a. | n.a. | n.a. | n.a. | n.a. | n.a. | n.a. | n.a. | n.a. | n.a. | n.a. | n.a. | n.a. | n.a. |  | n.a. | n.a. | n.a. | n.a. | n.a. | n.a. | n.a. | n.a. | n.a. | n.a. | n.a. | n.a. | n.a. |
| Shu X et al. |  | n.a. | n.a. | n.a. | n.a. | n.a. | n.a. | n.a. | n.a. | n.a. | n.a. | n.a. | n.a. | n.a. | n.a. | n.a. | n.a. | n.a. | n.a. | n.a. | n.a. | n.a. | n.a. | n.a. | n.a. | n.a. | n.a. | n.a. | n.a. | n.a. | n.a. | n.a. | n.a. |
| Silano et al. | n.a. | n.a. |  | n.a. | n.a. | n.a. | n.a. | n.a. | n.a. | n.a. | n.a. | n.a. | n.a. | n.a. | n.a. | n.a. | n.a. | n.a. | n.a. | n.a. | n.a. | n.a. | n.a. | n.a. | n.a. | n.a. | n.a. | n.a. | n.a. | n.a. | n.a. | n.a. | n.a. |
| Stockton et al. | n.a. | n.a. | n.a. |  | n.a. | n.a. | n.a. | n.a. | n.a. | n.a. | n.a. | n.a. | n.a. | n.a. | n.a. | n.a. |  | n.a. | n.a. | n.a. | n.a. | n.a. | n.a. | n.a. | n.a. | n.a. | n.a. | n.a. | n.a. | n.a. | n.a. | n.a. | n.a. |
| Swerdlow et al. |  | n.a. | n.a. | n.a. | n.a. | n.a. | n.a. | n.a. | n.a. | n.a. | n.a. | n.a. | n.a. | n.a. | n.a. | n.a. | n.a. | n.a. | n.a. | n.a. | n.a. | n.a. | n.a. | n.a. | n.a. | n.a. | n.a. | n.a. | n.a. | n.a. | n.a. | n.a. | n.a. |
| Tallbacka et al. | n.a. | n.a. | n.a. | n.a. | n.a. |  | n.a. | n.a. | n.a. | n.a. | n.a. | n.a. | n.a. | n.a. | n.a. | n.a. | n.a. | n.a. | n.a. | n.a. | n.a. | n.a. | n.a. | n.a. | n.a. | n.a. | n.a. | n.a. | n.a. | n.a. | n.a. | n.a. | n.a. |
| Thomas et al. | n.a. | n.a. | n.a. | n.a. | n.a. | n.a. |  | n.a. | n.a. | n.a. | n.a. | n.a. | n.a. | n.a. | n.a. | n.a. | n.a. | n.a. | n.a. | n.a. | n.a. | n.a. | n.a. | n.a. | n.a. | n.a. | n.a. | n.a. | n.a. | n.a. | n.a. | n.a. | n.a. |
| Van Daalen et al. | n.a. | n.a. | n.a. | n.a. | n.a. | n.a. | n.a. | n.a. | n.a. | n.a. | n.a. | n.a. |  | n.a. | n.a. | n.a. | n.a. | n.a. | n.a. | n.a. | n.a. | n.a. | n.a. | n.a. | n.a. | n.a. | n.a. | n.a. | n.a. | n.a. | n.a. | n.a. | n.a. |
| Viljaama et al. | n.a. | n.a. |  | n.a. | n.a. | n.a. | n.a. | n.a. | n.a. | n.a. |  | n.a. | n.a. | n.a. | n.a. | n.a. | n.a. | n.a. | n.a. | n.a. | n.a. | n.a. | n.a. | n.a. | n.a. | n.a. | n.a. | n.a. | n.a. | n.a. | n.a. | n.a. | n.a. |
| Weng et al. | n.a. | n.a. | n.a. | n.a. | n.a. | n.a. | n.a. |  | n.a. | n.a. | n.a. | n.a. | n.a. | n.a. | n.a. | n.a. | n.a. | n.a. | n.a. | n.a. | n.a. | n.a. | n.a. | n.a. | n.a. | n.a. | n.a. | n.a. | n.a. | n.a. | n.a. | n.a. | n.a. |
| Xin Long Lim et al. | n.a. | n.a. | n.a. | n.a. | n.a. | n.a. |  | n.a. | n.a. | n.a. | n.a. | n.a. | n.a. | n.a. | n.a. | n.a. | n.a. | n.a. | n.a. | n.a. | n.a. | n.a. | n.a. | n.a. | n.a. | n.a. | n.a. | n.a. | n.a. | n.a. | n.a. | n.a. | n.a. |
| Yamada et al. | n.a. | n.a. | n.a. | n.a. | n.a. | n.a. |  | n.a. | n.a. | n.a. | n.a. | n.a. | n.a. | n.a. | n.a. | n.a. | n.a. | n.a. | n.a. | n.a. | n.a. | n.a. | n.a. | n.a. | n.a. | n.a. | n.a. | n.a. | n.a. | n.a. | n.a. | n.a. | n.a. |
| Yoo et al. | n.a. | n.a. | n.a. | n.a. | n.a. | n.a. | n.a. | n.a. | n.a. | n.a. | n.a. | n.a. |  | n.a. | n.a. | n.a. | n.a. | n.a. | n.a. | n.a. | n.a. | n.a. | n.a. | n.a. | n.a. | n.a. | n.a. | n.a. | n.a. | n.a. | n.a. | n.a. | n.a. |

*T1DM= diabetes mellitus type 1; PA = pernicious anemia; CD = celiac disease; DM = dermatomyositis; PM = polymyositis; SLE = systemic lupus erythematosus; RA = rheumatoid arthritis; AS = ankylosing spondylitis; Ssc = systemic sclerosis; DH = dermatitis herpetiformis; AIV = autoimmune vasculitis; HT = Hashimoto’s thyroiditis; GD = Graves’ disease; IBD : inflammatory bowel disease; IM = inflammatory myopathies; PBC = primary biliary cirrhosis; UC = ulcerative colitis; AIP = autoimmune pancreatitis; MN = membranous nephropathy; AD = Addison’s disease; BD = Behçet disease; CRHD = chronic rheumatic heart disease; ITP = immune thrombocytopenic purpura; LS = localized scleroderma; MS = multiple sclerosis; MG = Myasthenia gravis; PR = polymyalgia rheumatica; PS = psoriasis; SR = sarcoidosis; IgG4RD = IgG4 related disease; DL = discoid lupus*

**Supplementary Figure 1. The standardized incidence ratios of gastric cancer in all examined autoimmune disorder – summarizing plot**

**
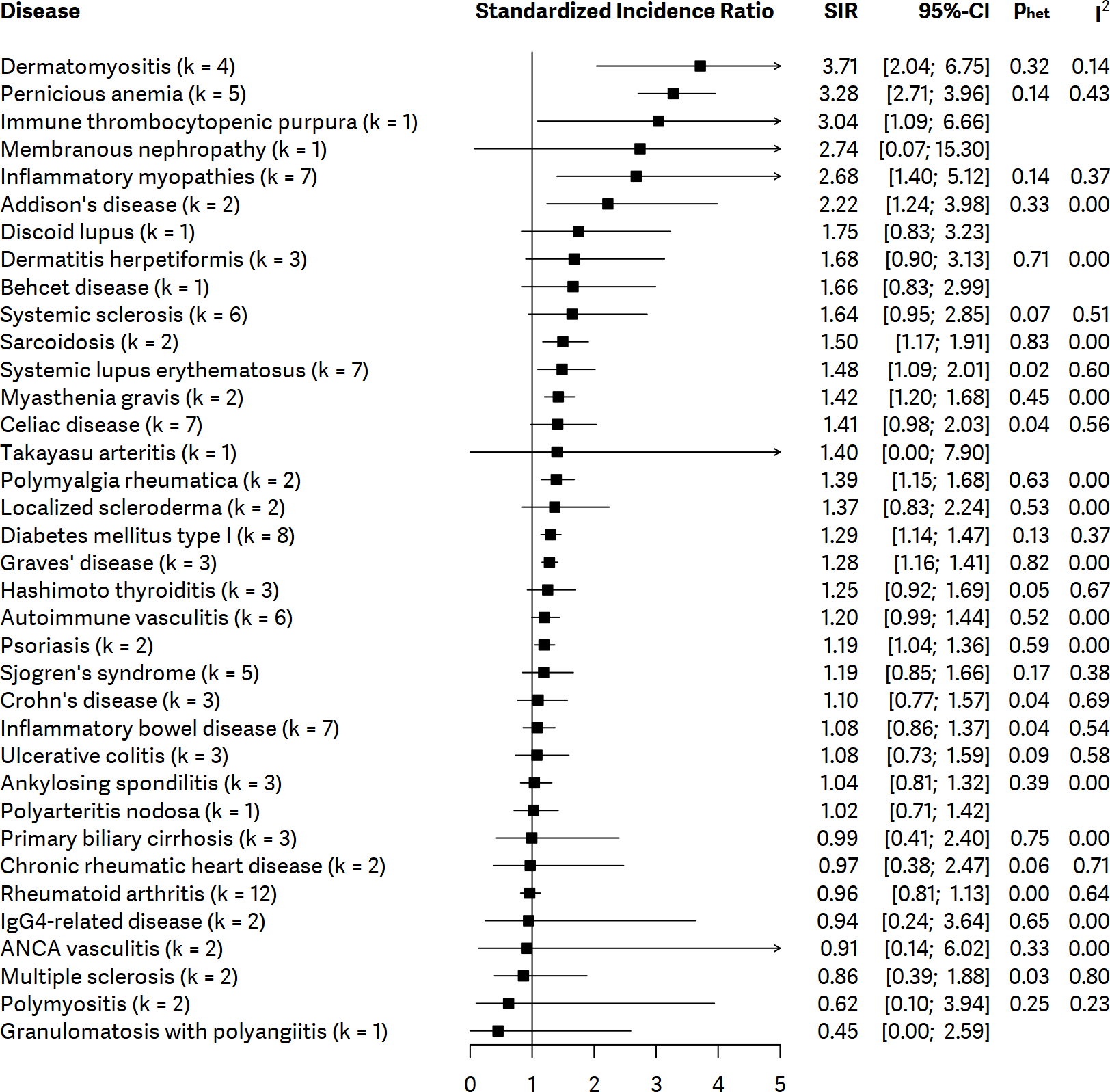
**

**Supplementary Figure 2. Forest plot showing standardized incidence ratios (SIR) of gastric cancer with 95% confidence interval (CI) for ankylosing spondylitis.**

**
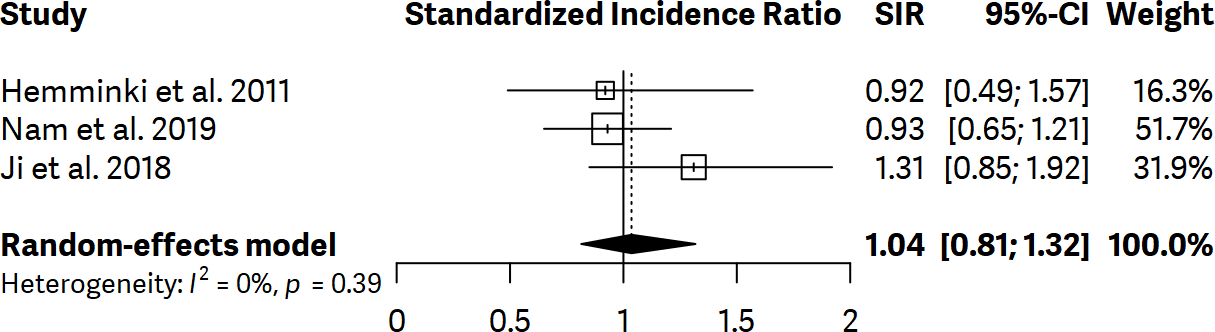
**

**Supplementary Figure 3. Forest plot showing standardized incidence ratios (SIR) of gastric cancer with 95% confidence interval (CI) for autoimmune vasculitis**

**
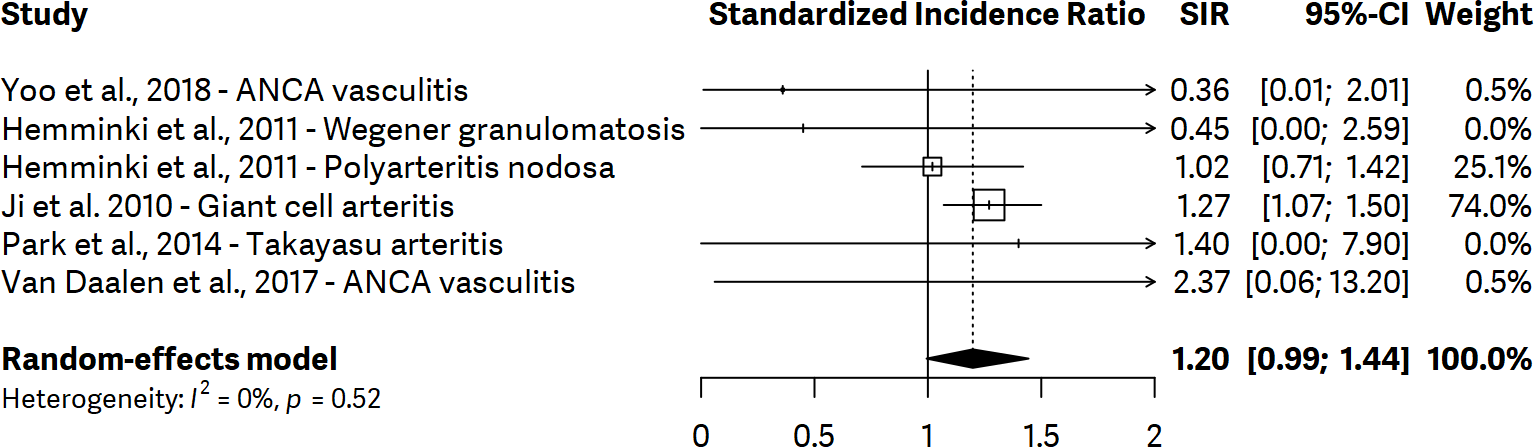
**

**Supplementary figure 4. Forest plot showing standardized incidence ratios (SIR) of gastric cancer with 95% confidence interval (CI) for celiac disease**

**
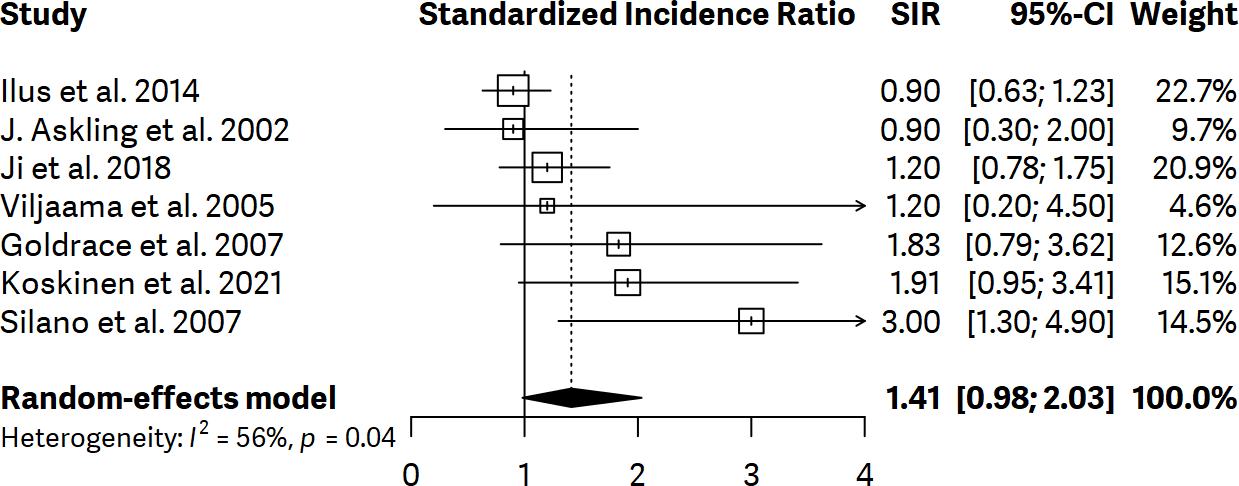
**

**Supplementary figure 5. Forest plot showing standardized incidence ratios (SIR) of gastric cancer with 95% confidence interval (CI) for Crohn’s disease**

**
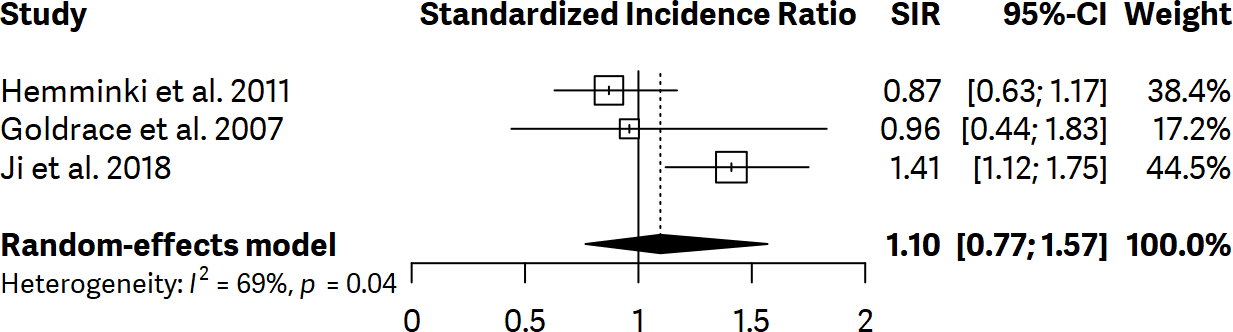
**

**Supplementary figure 6. Forest plot showing standardized incidence ratios (SIR) of gastric cancer with 95% confidence interval (CI) for dermatitis herpetiformis**

**
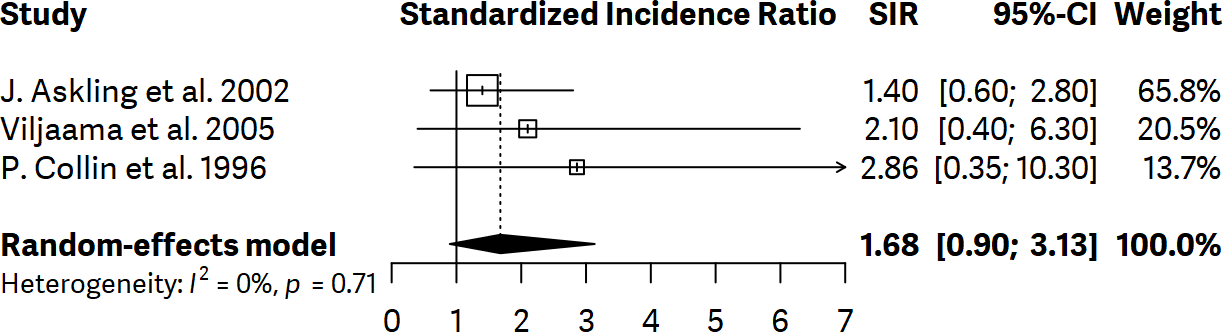
**

**Supplementary figure 7. Forest plot showing standardized incidence ratios (SIR) of gastric cancer with 95% confidence interval (CI) for dermatomyositis**

**
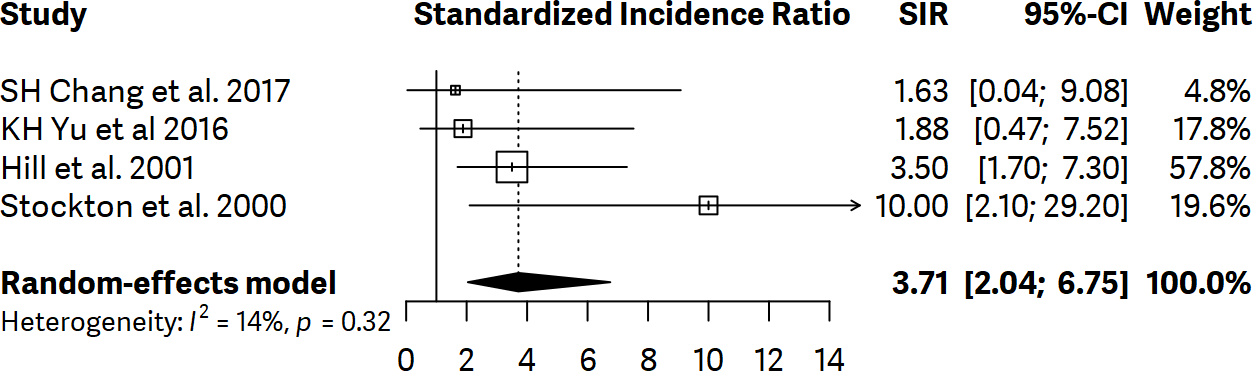
**

**Supplementary figure 8. Forest plot showing standardized incidence ratios (SIR) of gastric cancer with 95% confidence interval (CI) for diabetes mellitus type I.**

**
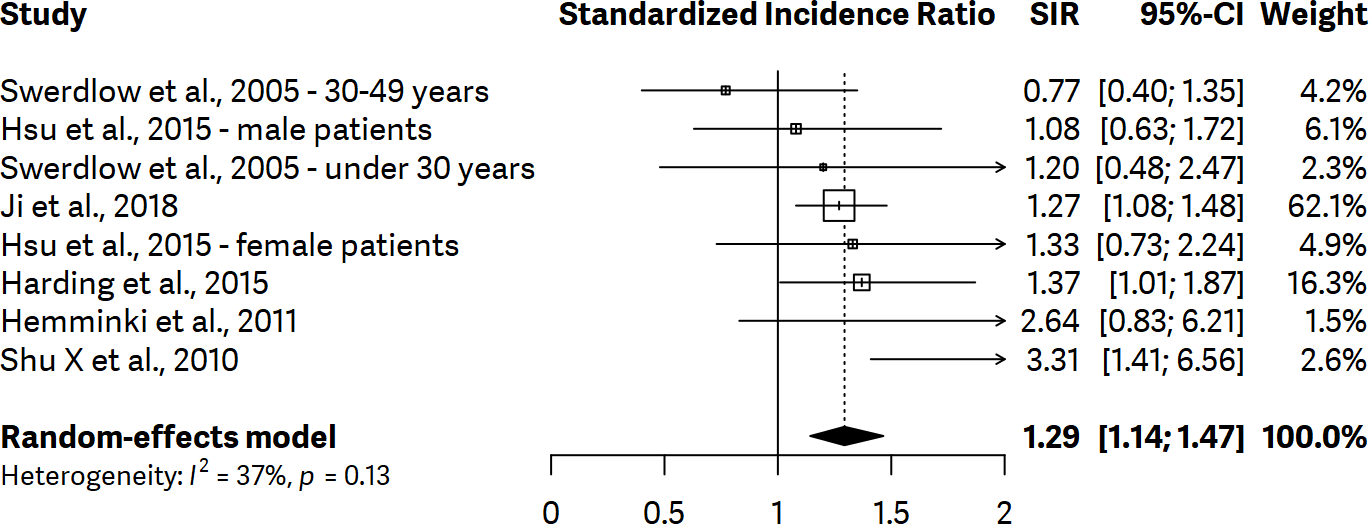
**

**Supplementary figure 9. Forest plot showing standardized incidence ratios (SIR) of gastric cancer with 95% confidence interval (CI) for Graves’ disease**

**
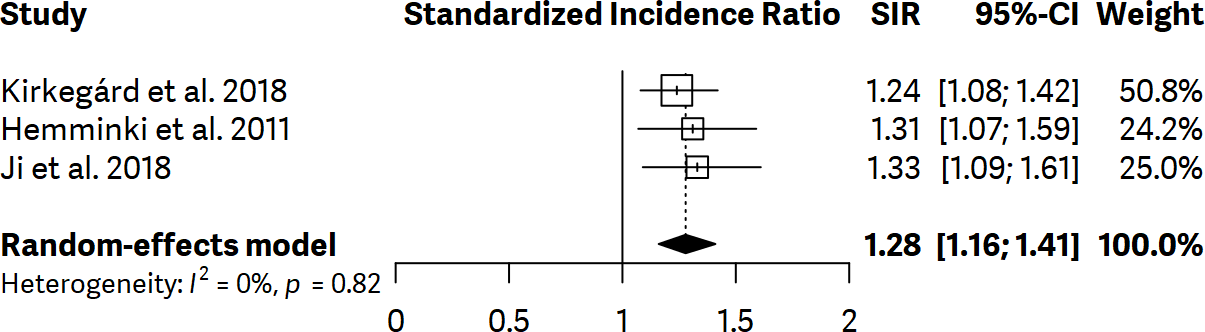
**

**Supplementary figure 10. Forest plot showing standardized incidence ratios (SIR) of gastric cancer with 95% confidence interval (CI) for Hashimoto’s thyroiditis**

**
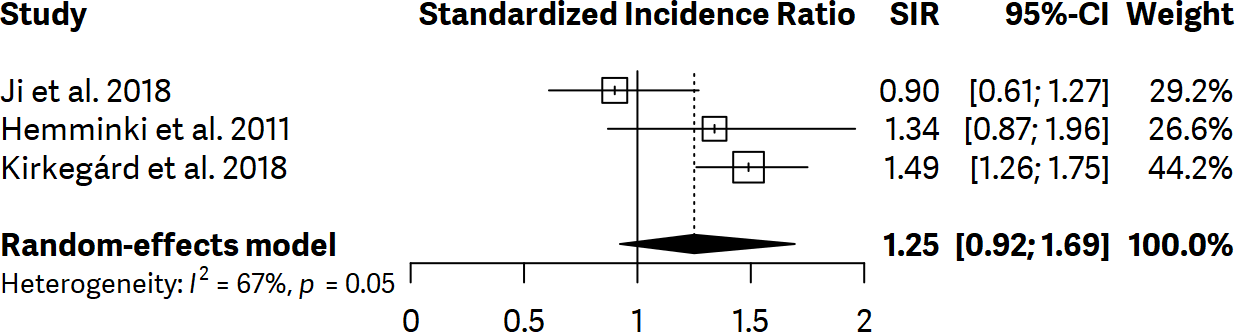
**

**Supplementary figure 11. Forest plot showing standardized incidence ratios (SIR) of gastric cancer with 95% confidence interval (CI) for inflammatory bowel disease**

**
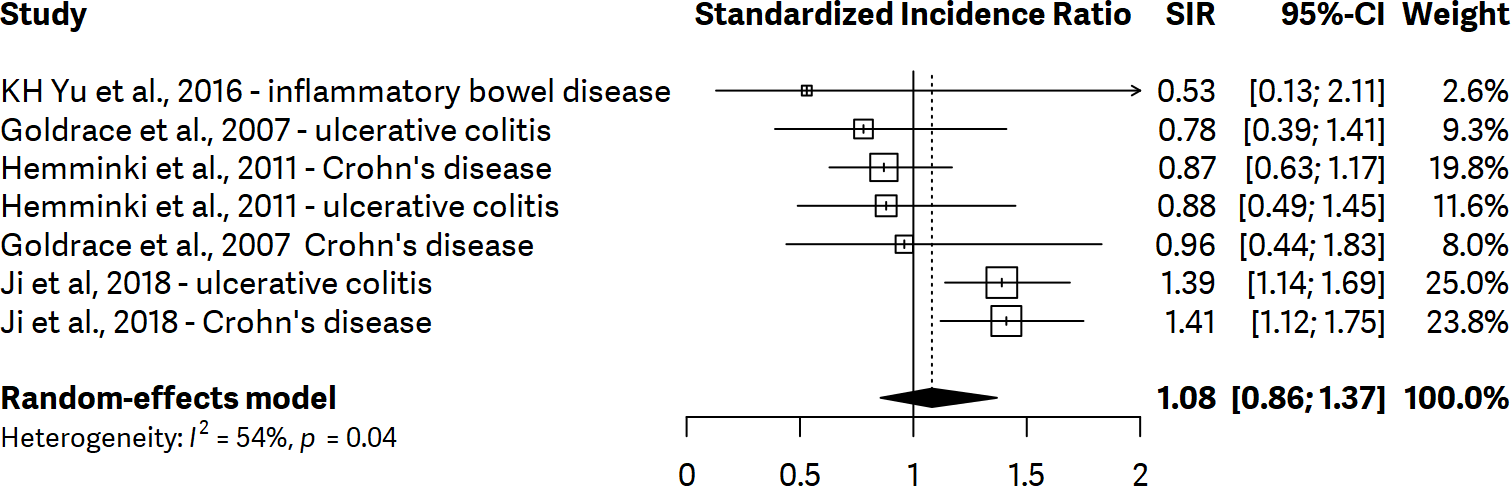
**

**Supplementary figure 12. Forest plot showing standardized incidence ratios (SIR) of gastric cancer with 95% confidence interval (CI) for inflammatory myopathies**

**
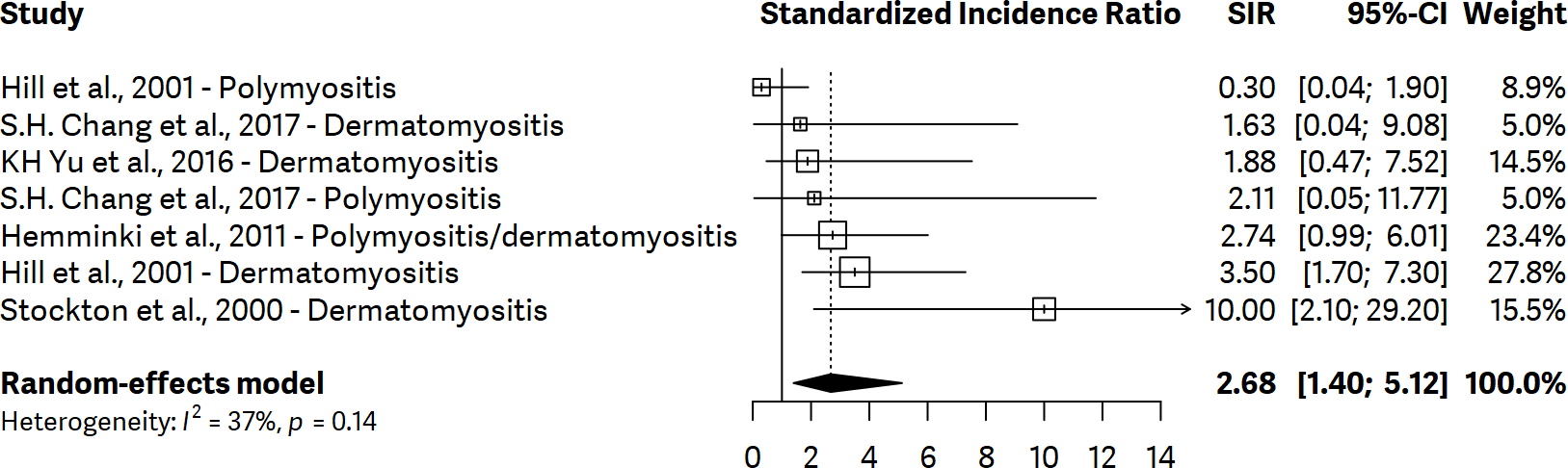
**

**Supplementary figure 13. Forest plot showing standardized incidence ratios (SIR) of gastric cancer with 95% confidence interval (CI) for pernicious anemia**

**
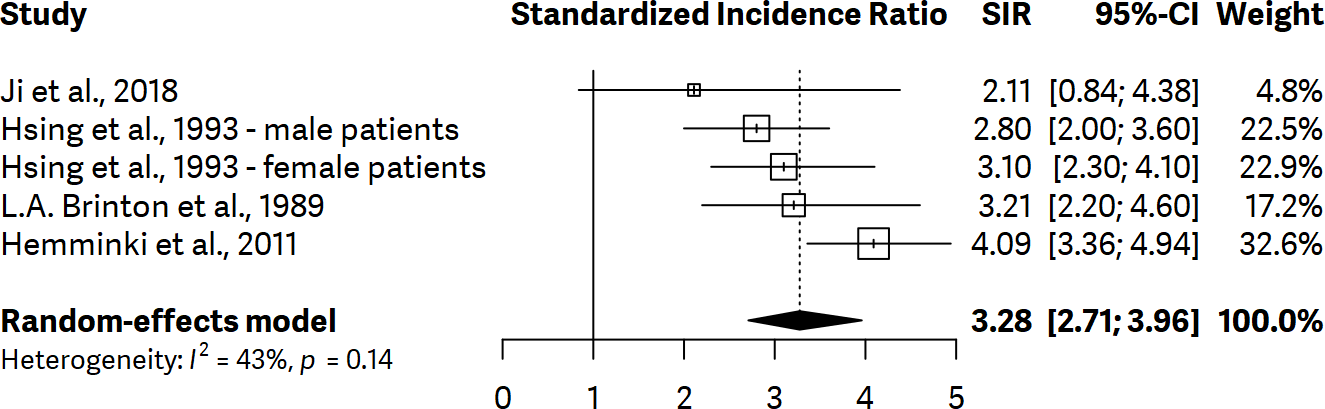
**

**Supplementary figure 14. Forest plot showing standardized incidence ratios (SIR) of gastric cancer with 95% confidence interval (CI) for primary biliary cirrhosis**

**
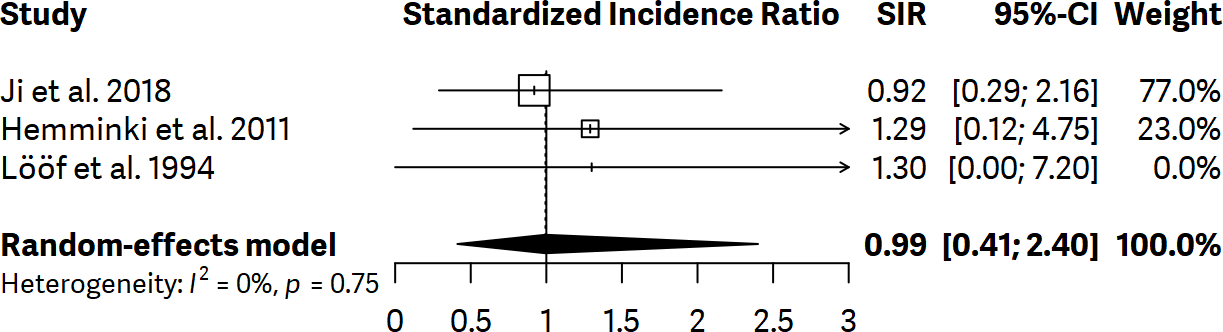
**

**Supplementary figure 15. Forest plot showing standardized incidence ratios (SIR) of gastric cancer with 95% confidence interval (CI) for rheumatoid arthritis**

**
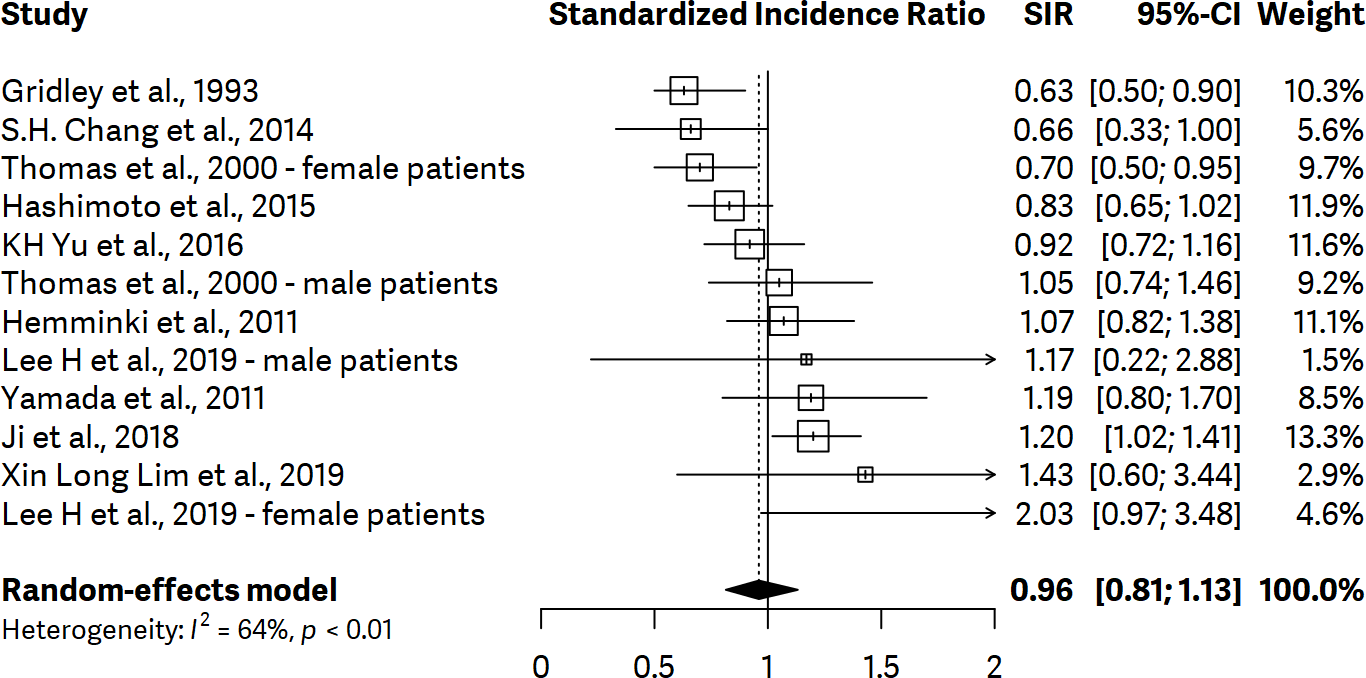
**

**Supplementary figure 16. Forest plot showing standardized incidence ratios (SIR) of gastric cancer with 95% confidence interval (CI) for Sjogren’s syndrome**

**
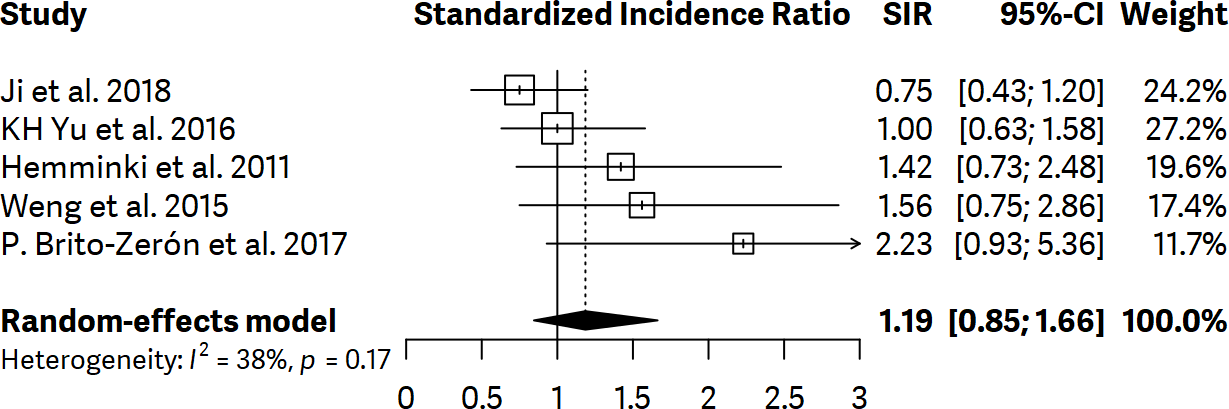
**

**Supplementary figure 17. Forest plot showing standardized incidence ratios (SIR) of gastric cancer with 95% confidence interval (CI) for systemic lupus erythematosus**

**
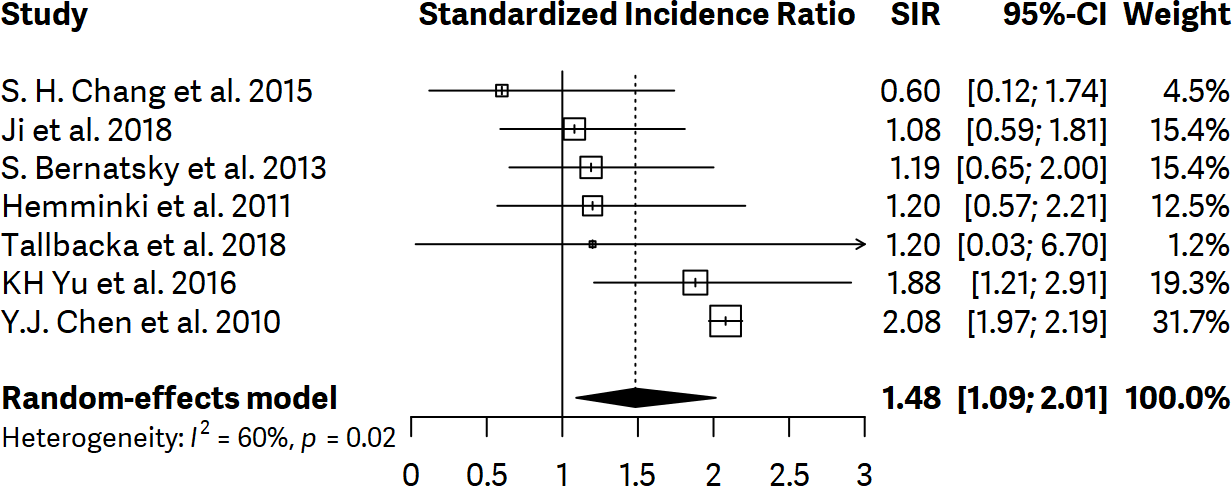
**

**Supplementary figure 18. Forest plot showing standardized incidence ratios (SIR) of gastric cancer with 95% confidence interval (CI) for systemic sclerosis**

**
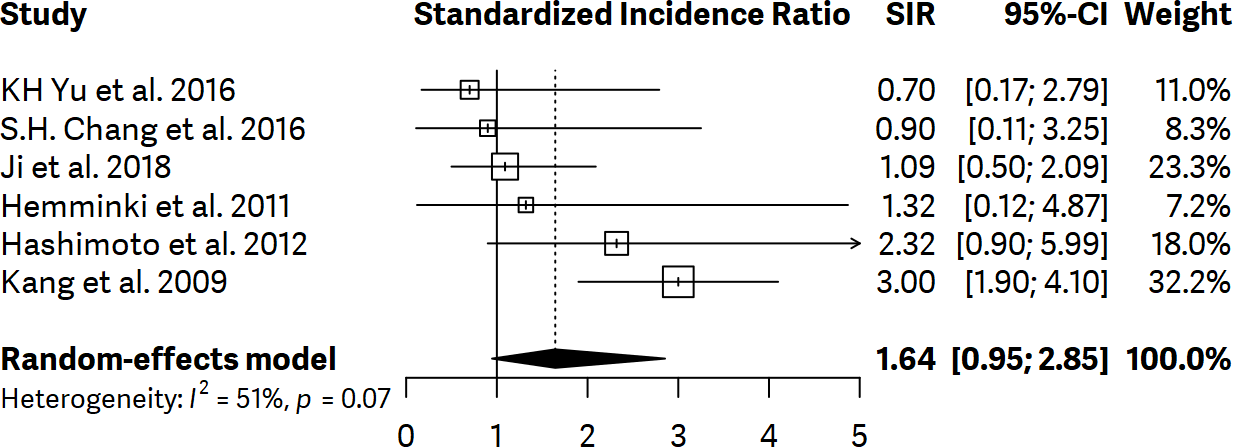
**

**Supplementary figure 19. Forest plot showing standardized incidence ratios (SIR) of gastric cancer with 95% confidence interval (CI) for ulcerative colitis**

**
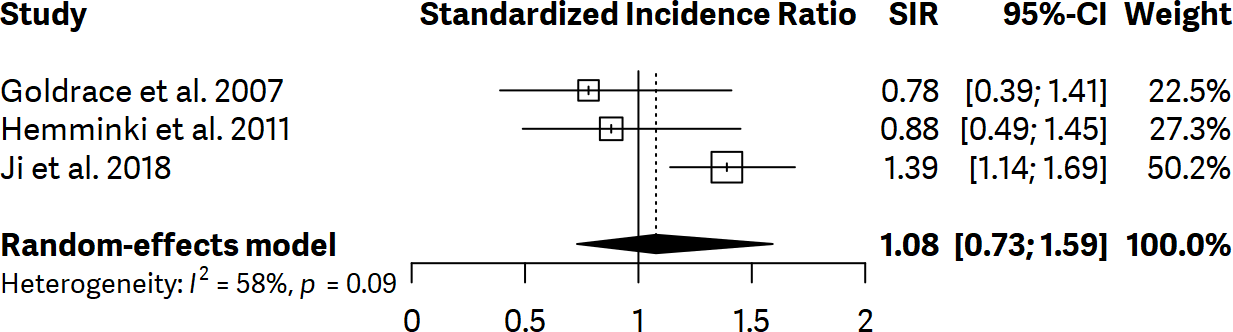
**

**Supplementary Figure 20. Forest plot showing standardized incidence ratios (SIR) of gastric cancer with 95% confidence interval (CI) for Addison’s disease**

**
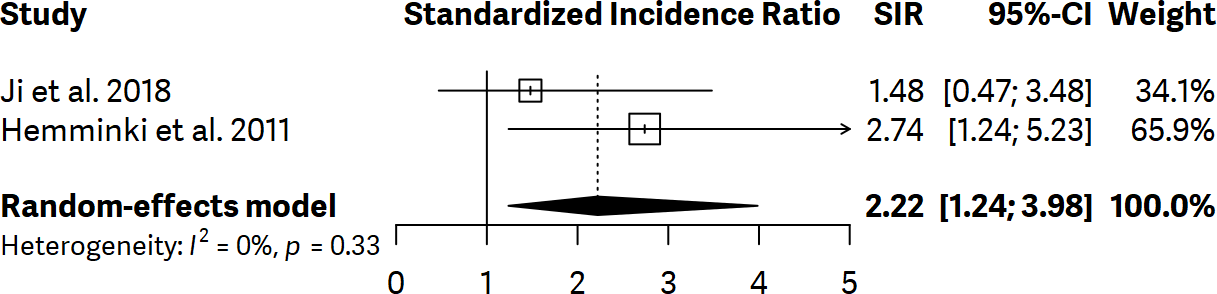
**

**Supplementary Figure 21. Forest plot showing standardized incidence ratios (SIR) of gastric cancer with 95% confidence interval (CI) for autoimmune pancreatitis**

**
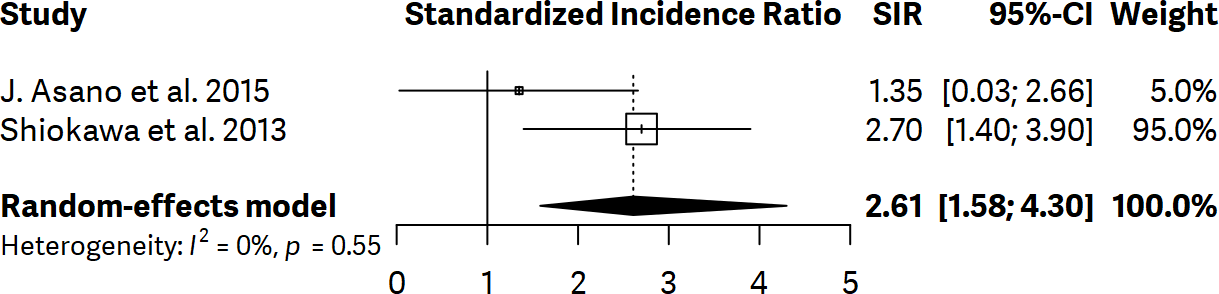
**

**Supplementary figure 22. Forest plot showing standardized incidence ratios (SIR) of gastric cancer with 95% confidence interval (CI) for ANCA vasculitis**

**
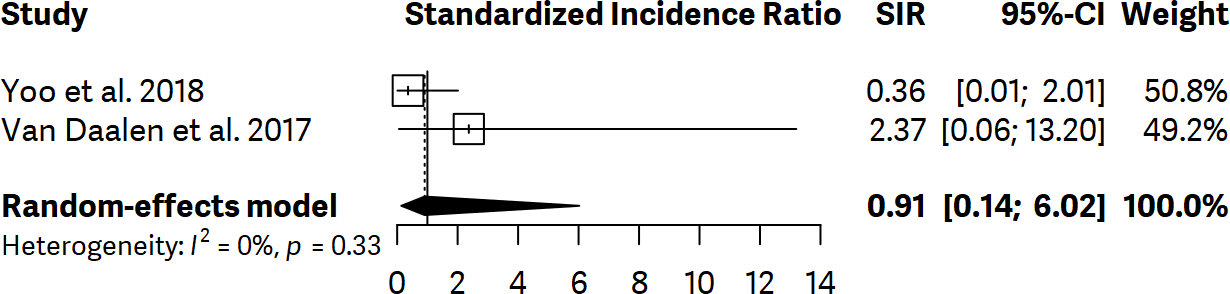
**

**Supplementary figure 23. Forest plot showing standardized incidence ratios (SIR) of gastric cancer with 95% confidence interval (CI) for Behçet's disease**

**
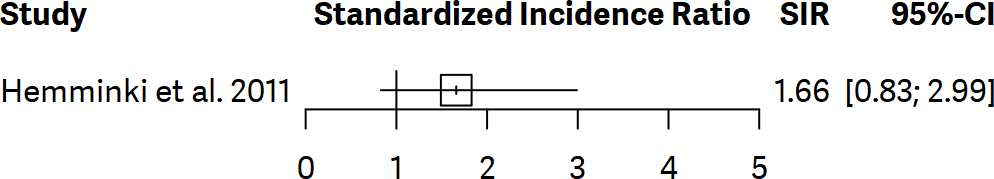
**

**Supplementary figure 24. Forest plot showing standardized incidence ratios (SIR) of gastric cancer with 95% confidence interval (CI) for chronic rheumatic heart disease**

**
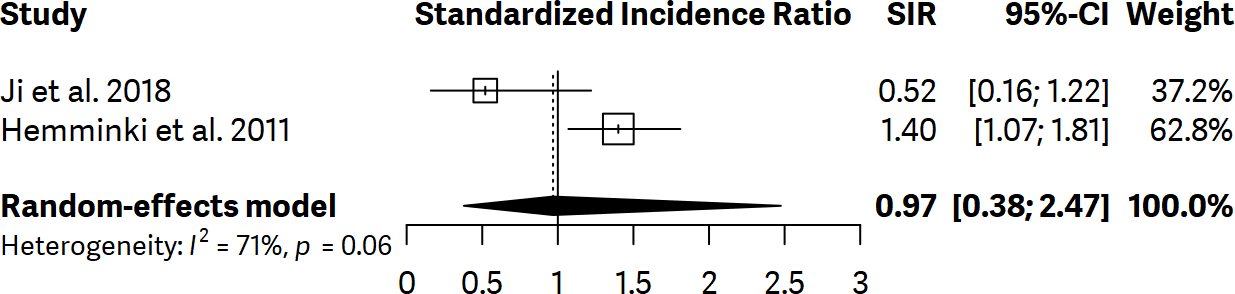
**

**Supplementary figure 25. Forest plot showing standardized incidence ratios (SIR) of gastric cancer with 95% confidence interval (CI) for discoid lupus**

**
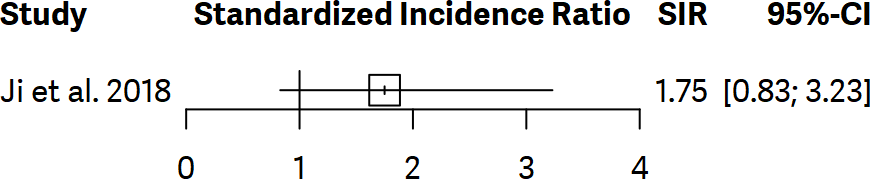
**

**Supplementary figure 26. Forest plot showing standardized incidence ratios (SIR) of gastric cancer with 95% confidence interval (CI) for granulomatosis with polyangiitis**

**
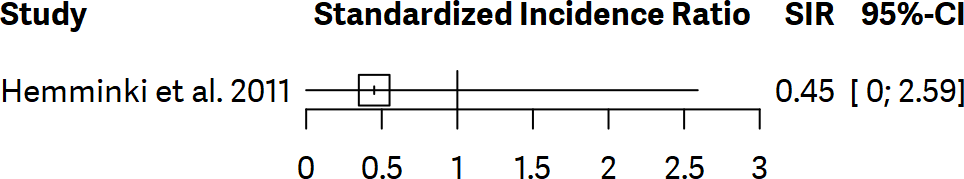
**

**Supplementary figure 27. Forest plot showing standardized incidence ratios (SIR) of gastric cancer with 95% confidence interval (CI) for IgG4-related disase**

**
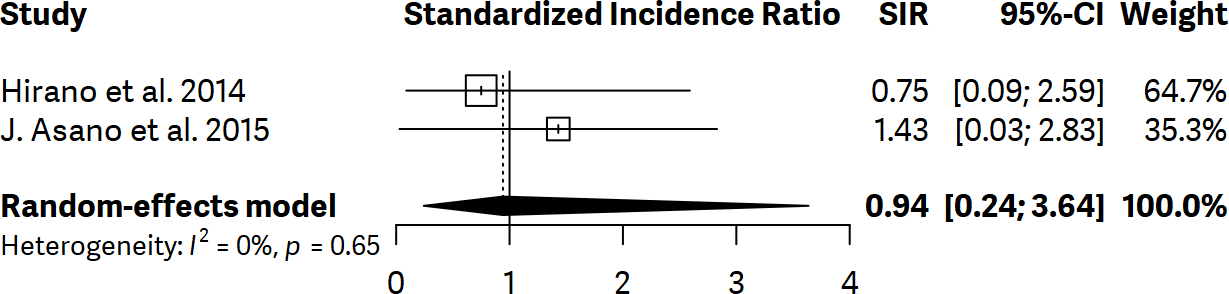
**

**Supplementary figure 28. Forest plot showing standardized incidence ratios (SIR) of gastric cancer with 95% confidence interval (CI) for immune thrombocytopenic purpura**

**
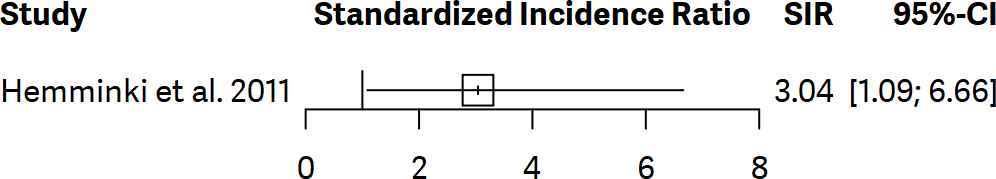
**

**Supplementary figure 29. Forest plot showing standardized incidence ratios (SIR) of gastric cancer with 95% confidence interval (CI) for localized scleroderma**

**
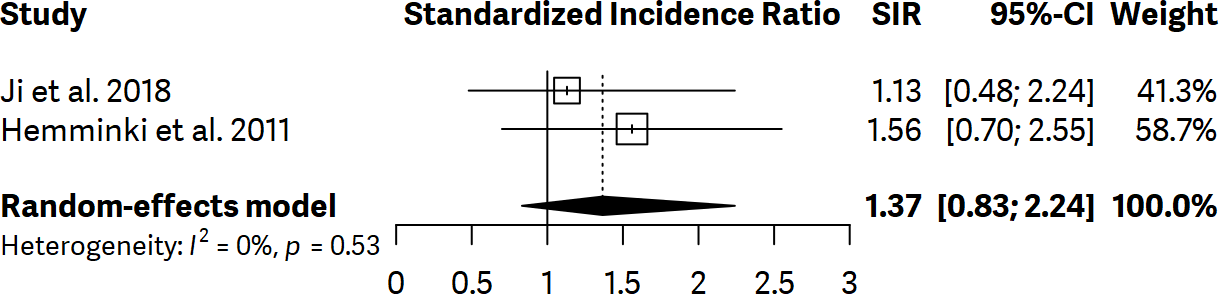
**

**Supplementary figure 30. Forest plot showing standardized incidence ratios (SIR) of gastric cancer with 95% confidence interval (CI) for membranous nephropathy**

**
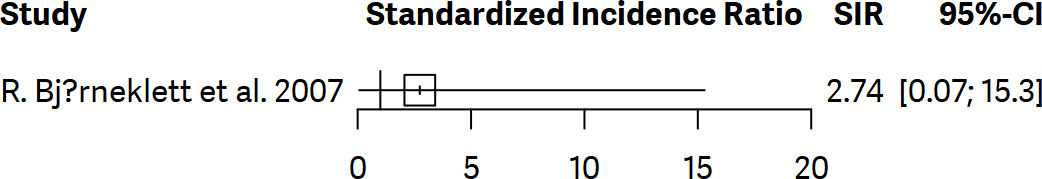
**

**Supplementary figure 31. Forest plot showing standardized incidence ratios (SIR) of gastric cancer with 95% confidence interval (CI) for multiple sclerosis**

**
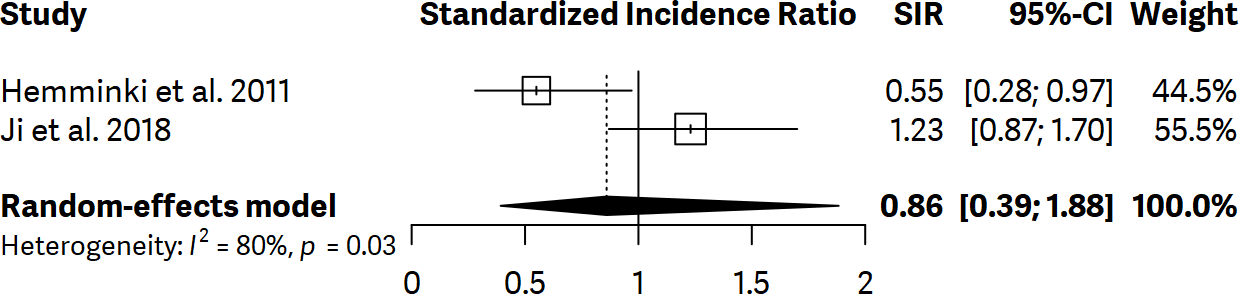
**

**Supplementary figure 32. Forest plot showing standardized incidence ratios (SIR) of gastric cancer with 95% confidence interval (CI) for myasthenia gravis**

**
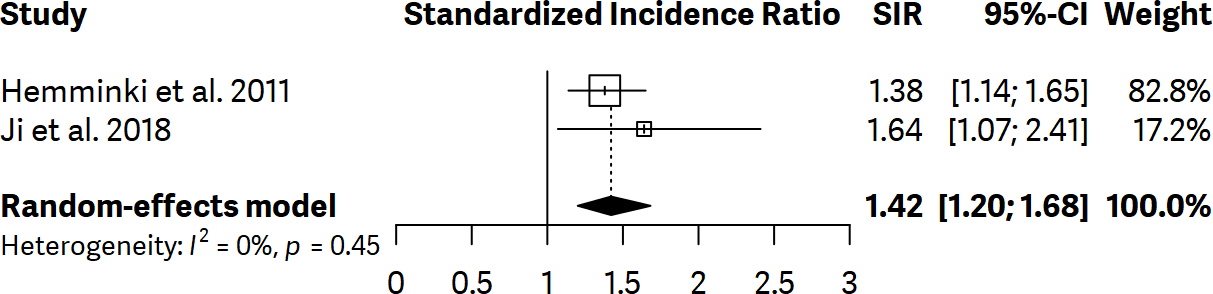
**

**Supplementary figure 33. Forest plot showing standardized incidence ratios (SIR) of gastric cancer with 95% confidence interval (CI) for polyarteritis nodosa**

**
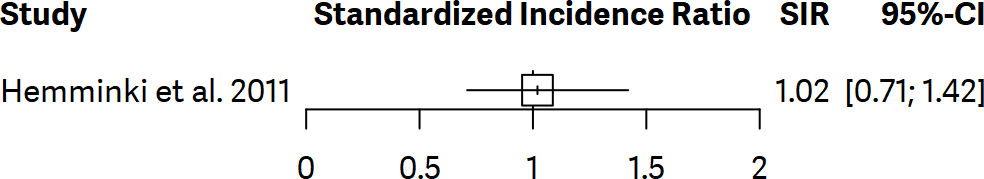
**

**Supplementary figure 34. Forest plot showing standardized incidence ratios (SIR) of gastric cancer with 95% confidence interval (CI) for polymyalgia rheumatica**

**
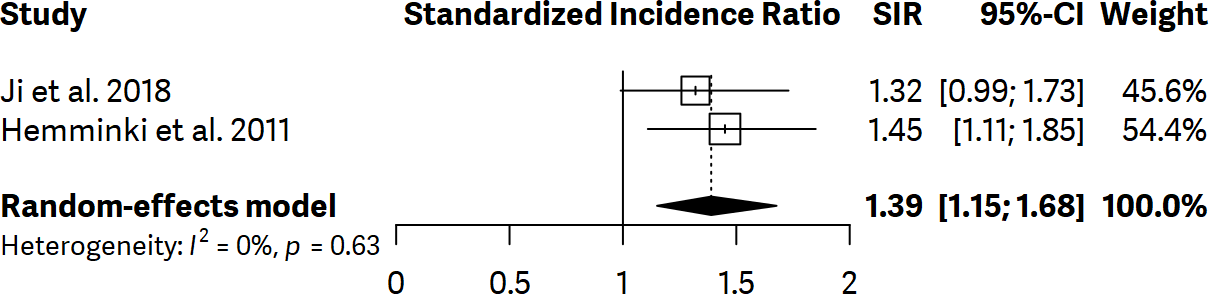
**

**Supplementary figure 35. Forest plot showing standardized incidence ratios (SIR) of gastric cancer with 95% confidence interval (CI) for polymyositis**

**
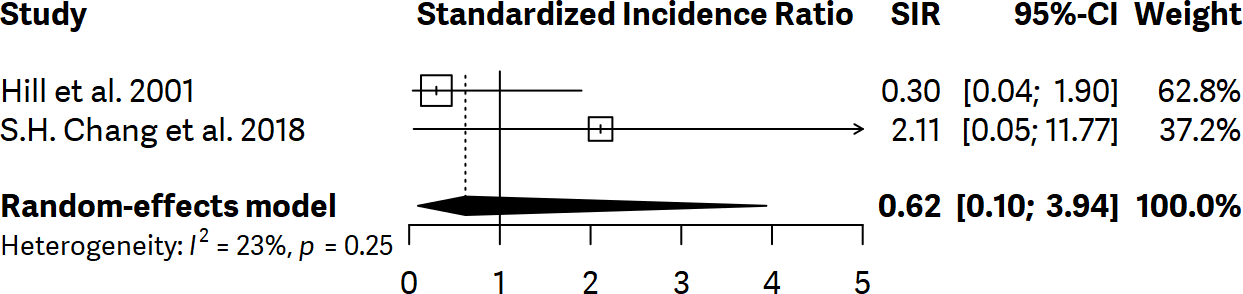
**

**Supplementary figure 36. Forest plot showing standardized incidence ratios (SIR) of gastric cancer with 95% confidence interval (CI) for psoriasis**

**
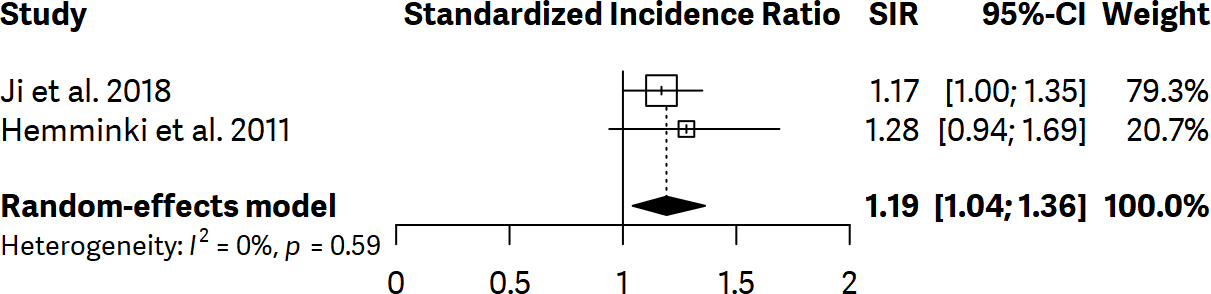
**

**Supplementary figure 37. Forest plot showing standardized incidence ratios (SIR) of gastric cancer with 95% confidence interval (CI) for sarcoidosis**

**
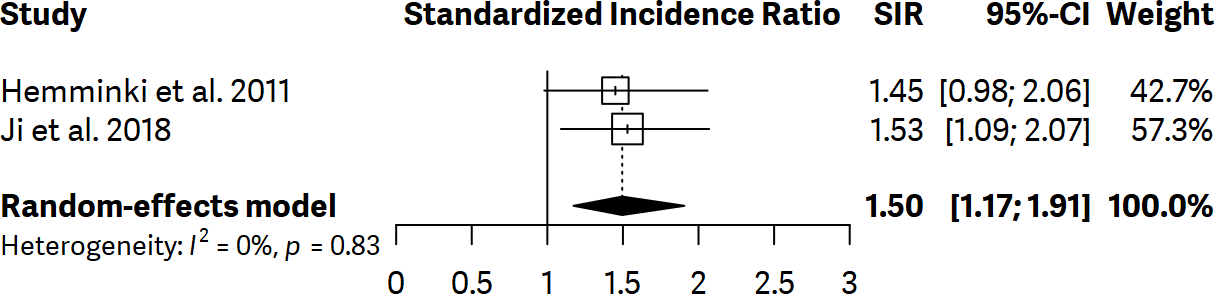
**

**Supplementary figure 38. Forest plot showing standardized incidence ratios (SIR) of gastric cancer with 95% confidence interval (CI) for Takayasu arteritis**

**
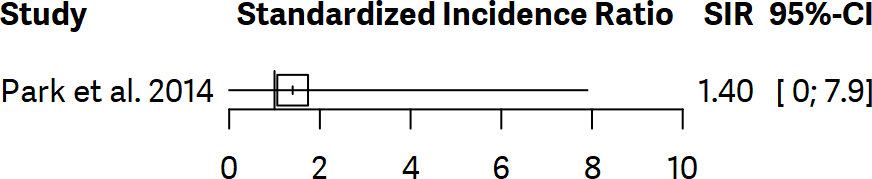
**

**Supplementary figure 39. Forest plot showing subgroup analysis regarding standardized incidence ratios (SIR) of gastric cancer with 95% confidence interval (CI) for diabetes mellitus type 1 based on gender.**

**
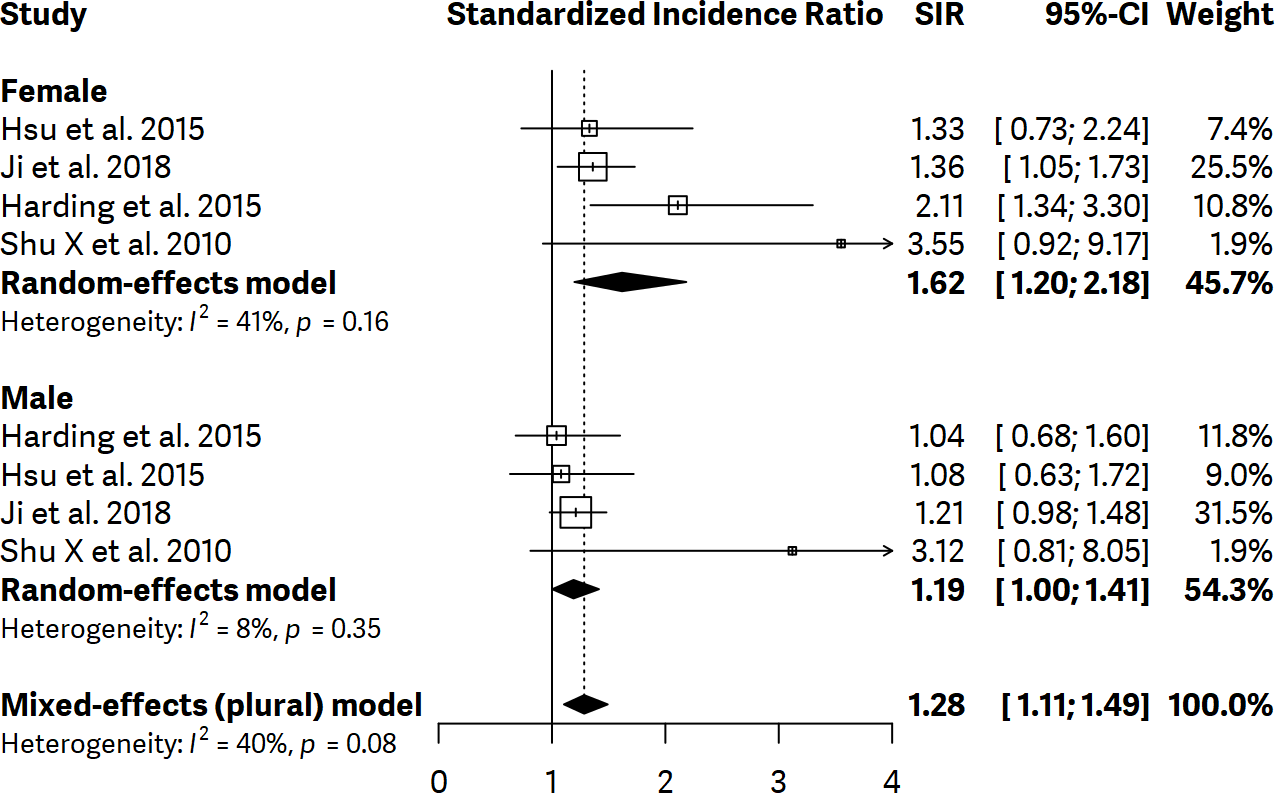
**

**Supplementary figure 40. Forest plot showing subgroup analysis regarding standardized incidence ratios (SIR) of gastric cancer with 95% confidence interval (CI) for rheumatoid arthritis based on gender.**

**
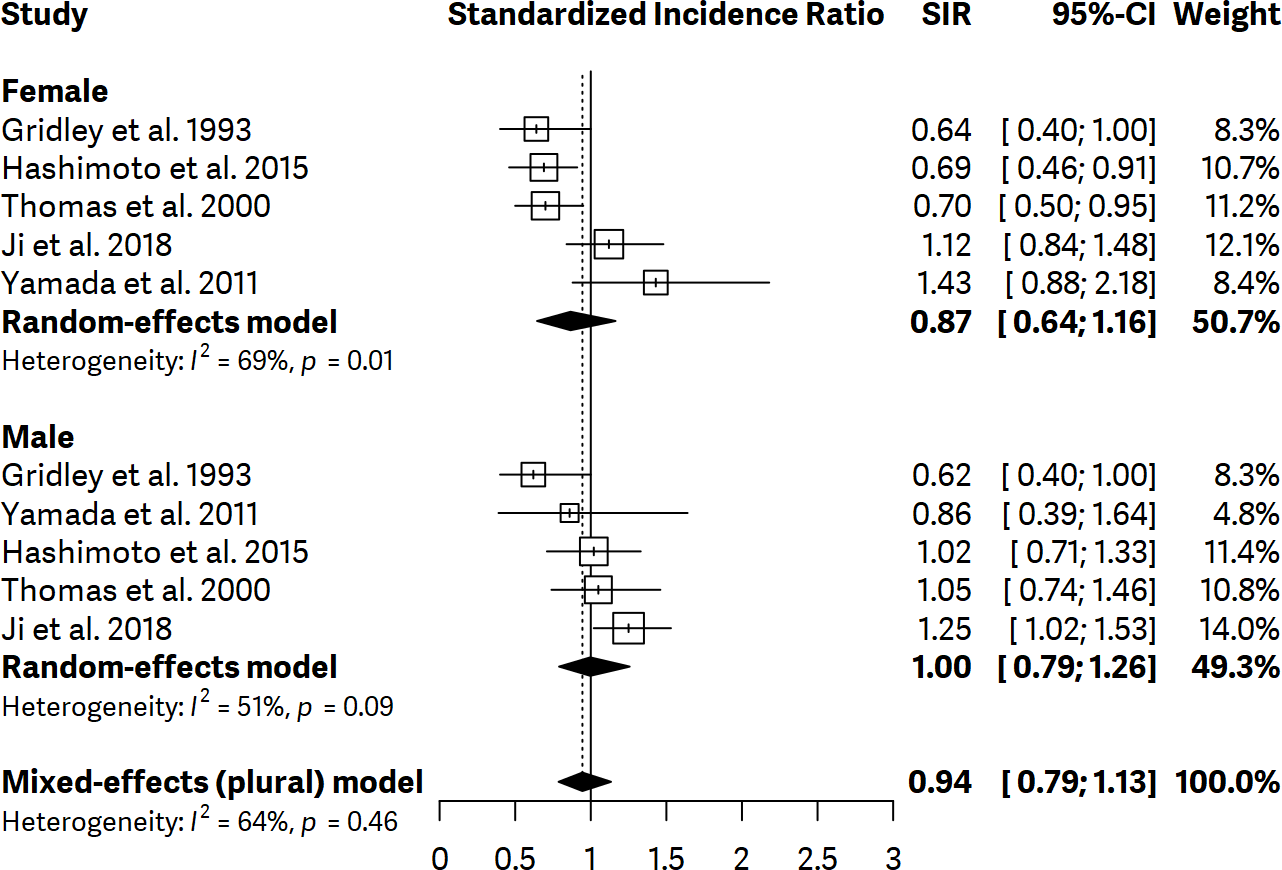
**

**Supplementary figure 41. Forest plot showing subgroup analysis regarding standardized incidence ratios (SIR) of gastric cancer with 95% confidence interval (CI) for ankylosing spondylitis based on high-, or low-incidence countries of gastric cancer.**

**
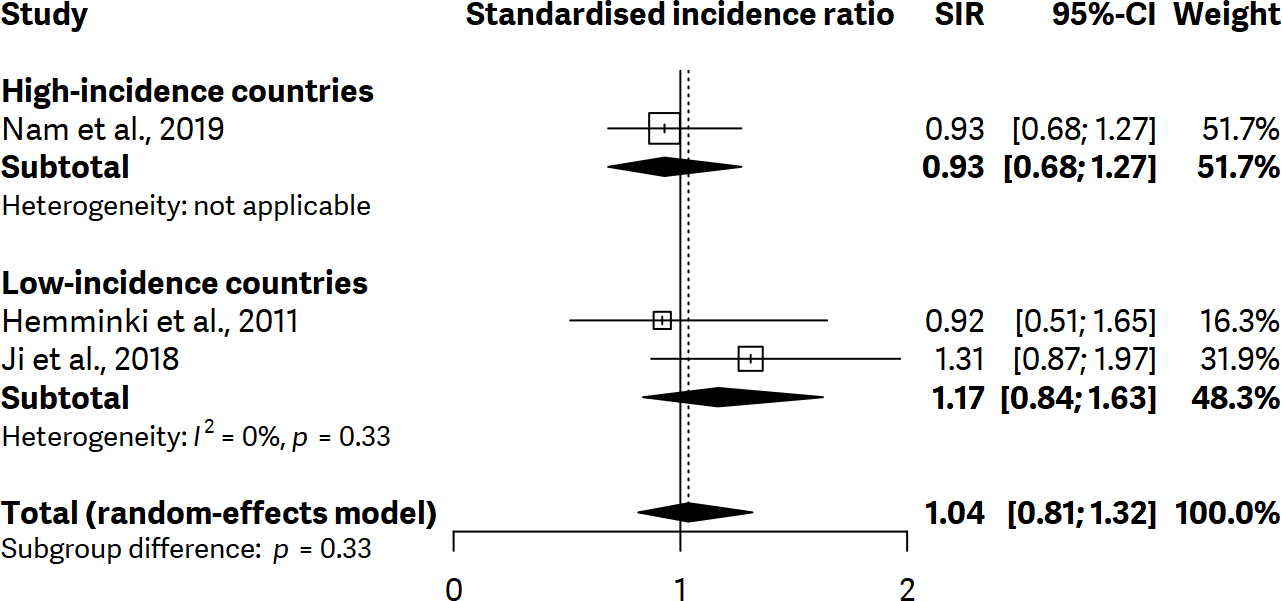
**

**Supplementary figure 42. Forest plot showing subgroup analysis regarding standardized incidence ratios (SIR) of gastric cancer with 95% confidence interval (CI) for celiac disease based on high-, or low-incidence countries of gastric cancer.**

**
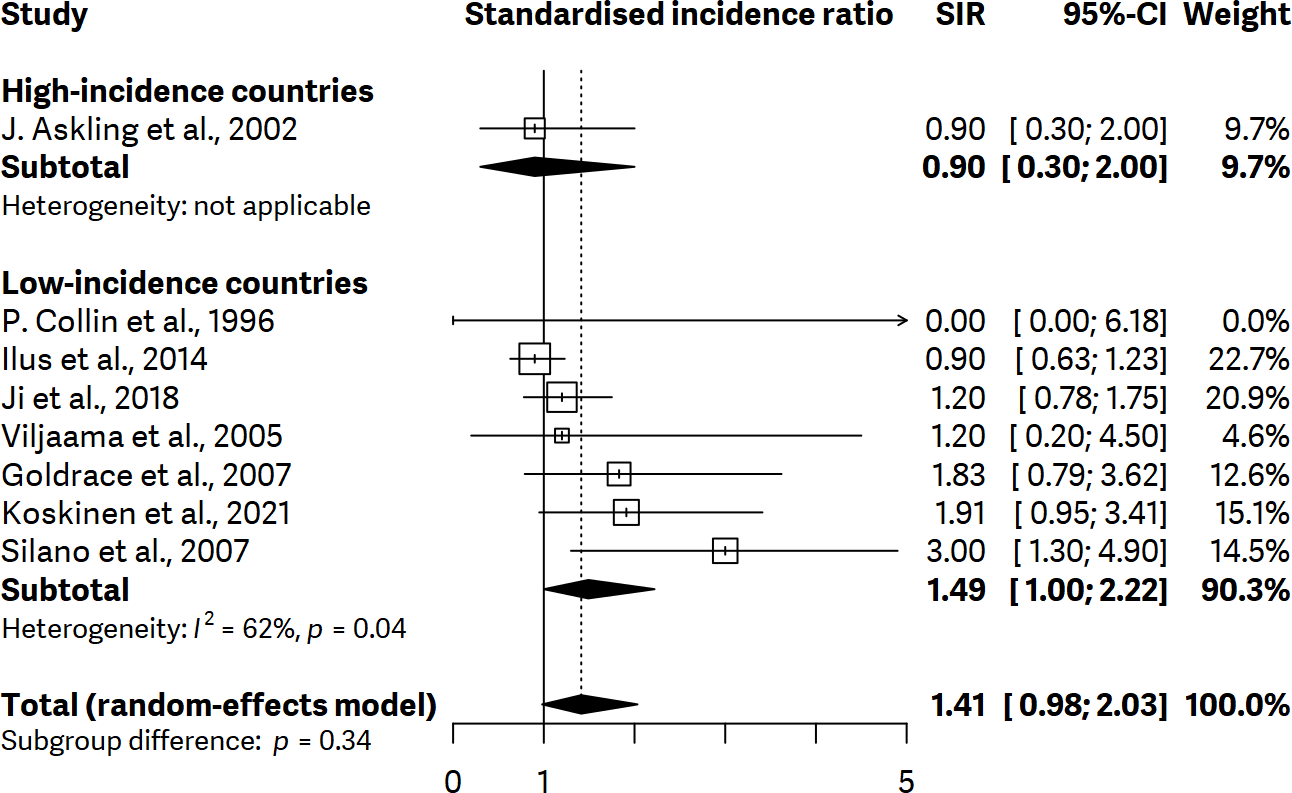
**

**Supplementary figure 43. Forest plot showing subgroup analysis regarding standardized incidence ratios (SIR) of gastric cancer with 95% confidence interval (CI) for dermatomyositis based on high-, or low-incidence countries of gastric cancer.**

**
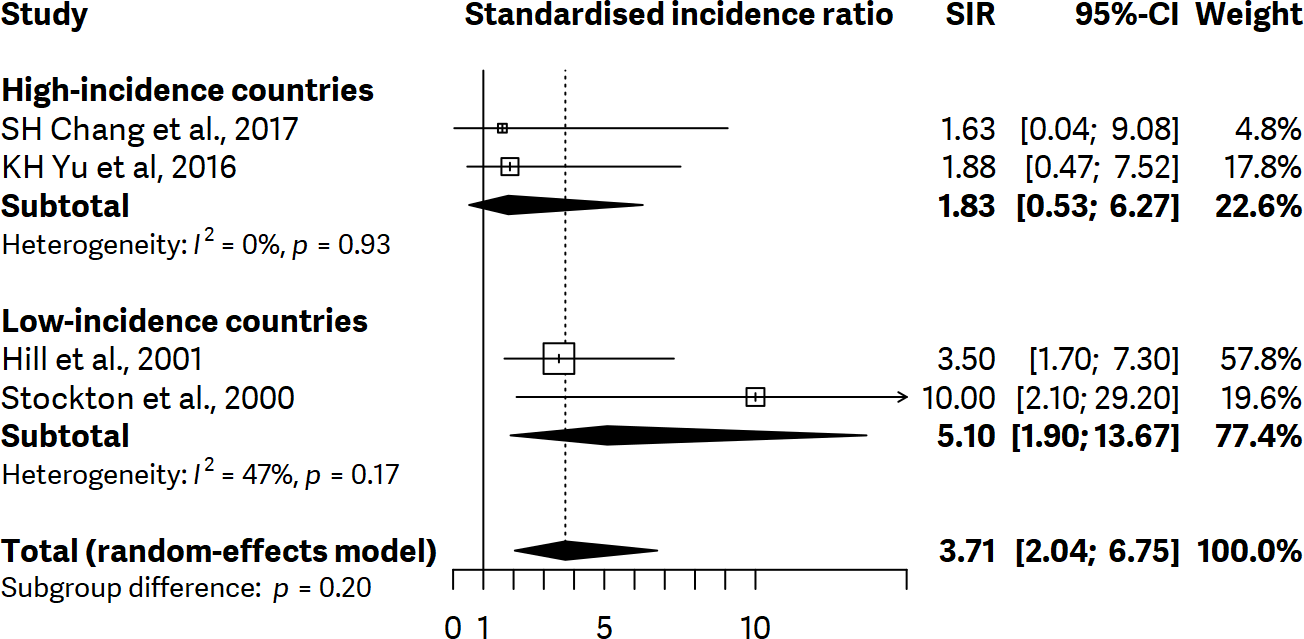
**

**Supplementary figure 44. Forest plot showing subgroup analysis regarding standardized incidence ratios (SIR) of gastric cancer with 95% confidence interval (CI) for dermatitis herpetiformis based on high-, or low-incidence countries of gastric cancer.**

**
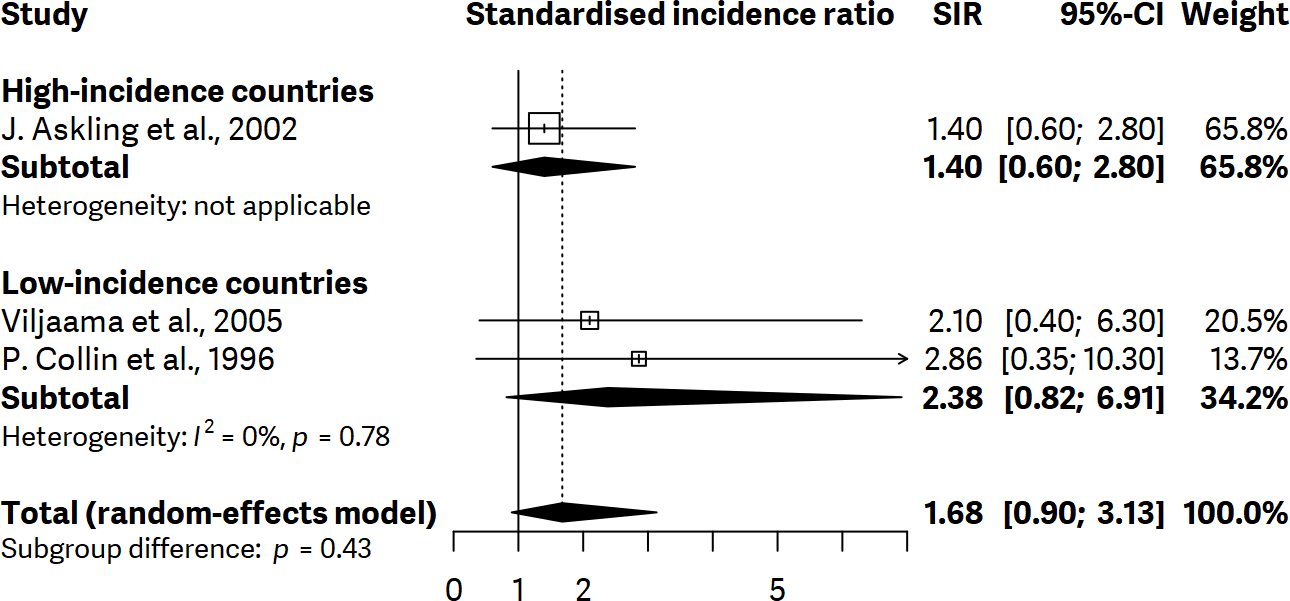
**

**Supplementary figure 45. Forest plot showing subgroup analysis regarding standardized incidence ratios (SIR) of gastric cancer with 95% confidence interval (CI) for diabetes mellitus type I based on high-, or low-incidence countries of gastric cancer.**

**
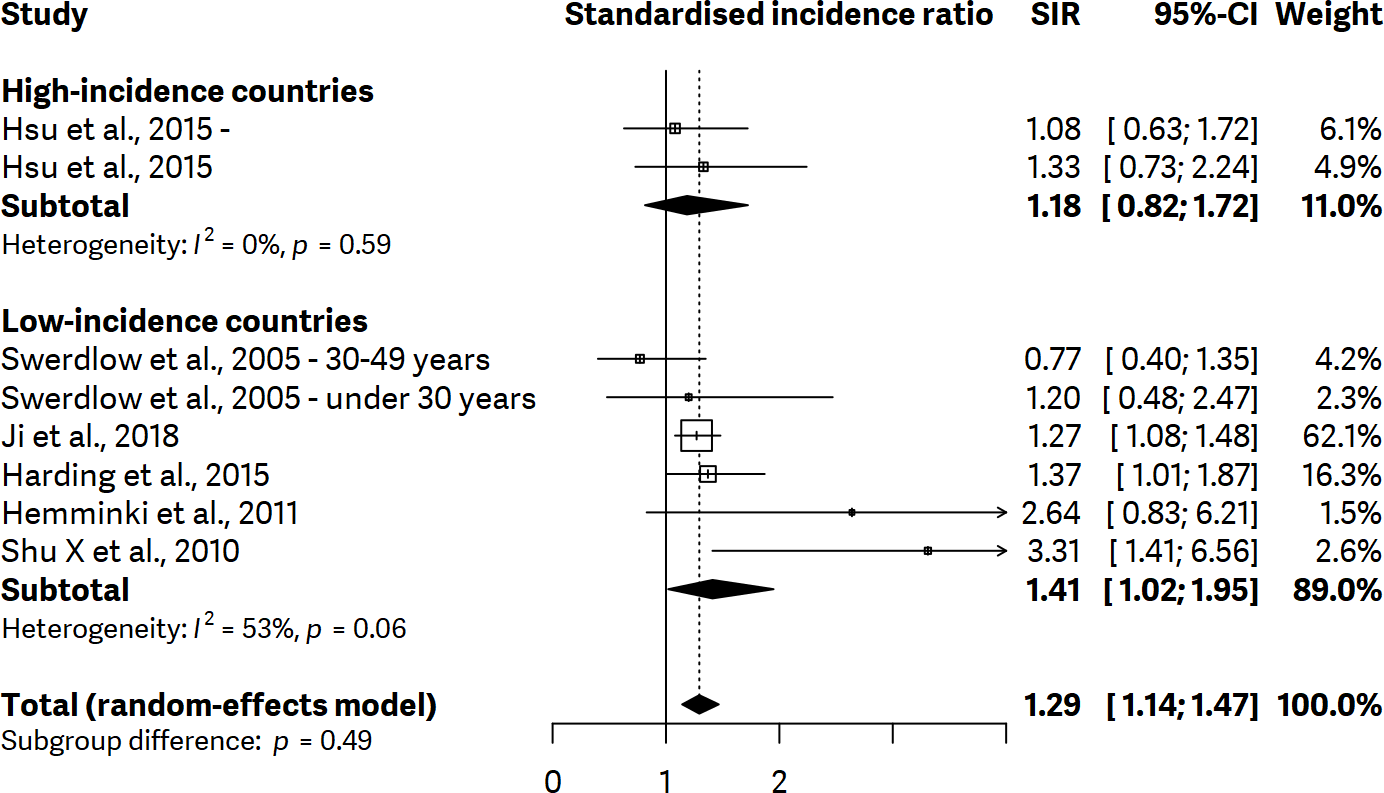
**

**Supplementary figure 46. Forest plot showing subgroup analysis regarding standardized incidence ratios (SIR) of gastric cancer with 95% confidence interval (CI) for autoimmune vasculitis based on high-, or low-incidence countries of gastric cancer.**

**
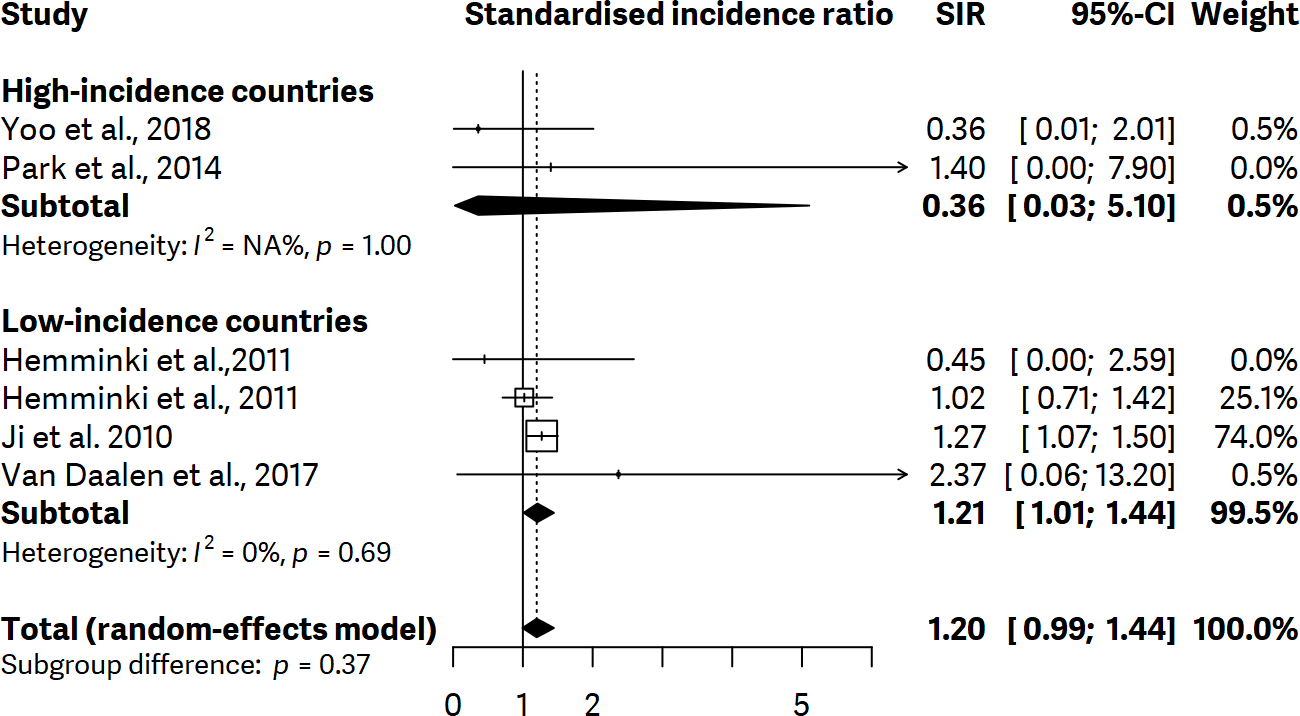
**

**Supplementary figure 47. Forest plot showing subgroup analysis regarding standardized incidence ratios (SIR) of gastric cancer with 95% confidence interval (CI) for Graves’ disease based on high-, or low-incidence countries of gastric cancer.**

**
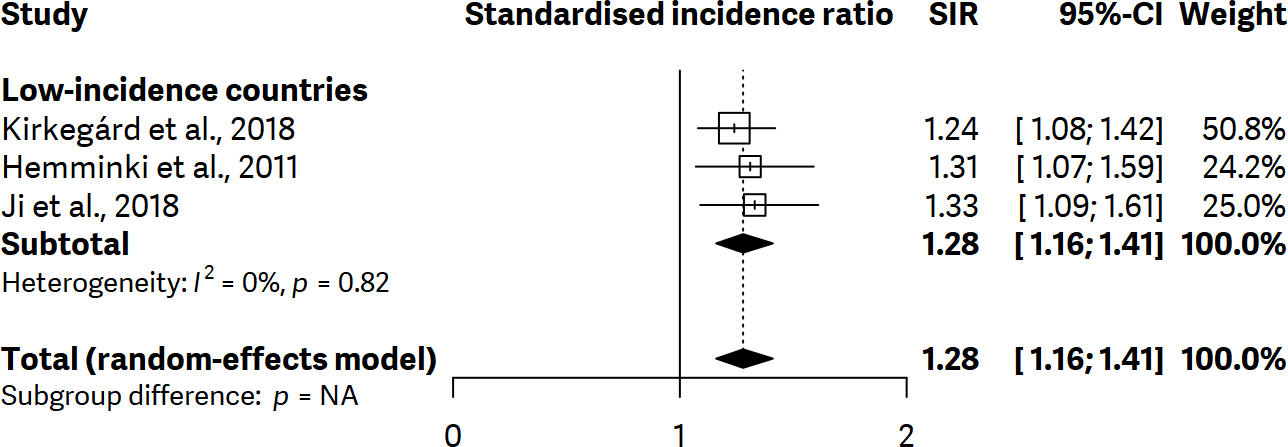
**

**Supplementary figure 48. Forest plot showing subgroup analysis regarding standardized incidence ratios (SIR) of gastric cancer with 95% confidence interval (CI) for Hashimoto thyroiditis based on high-, or low-incidence countries of gastric cancer.**

**
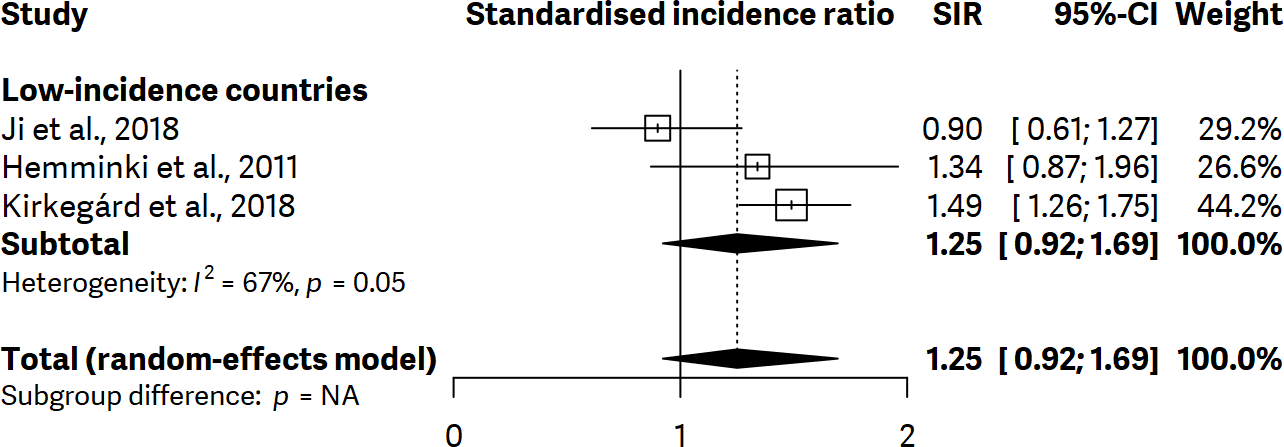
**

**Supplementary figure 49. Forest plot showing subgroup analysis regarding standardized incidence ratios (SIR) of gastric cancer with 95% confidence interval (CI) for pernicious anaemia based on high-, or low-incidence countries of gastric cancer.**

**
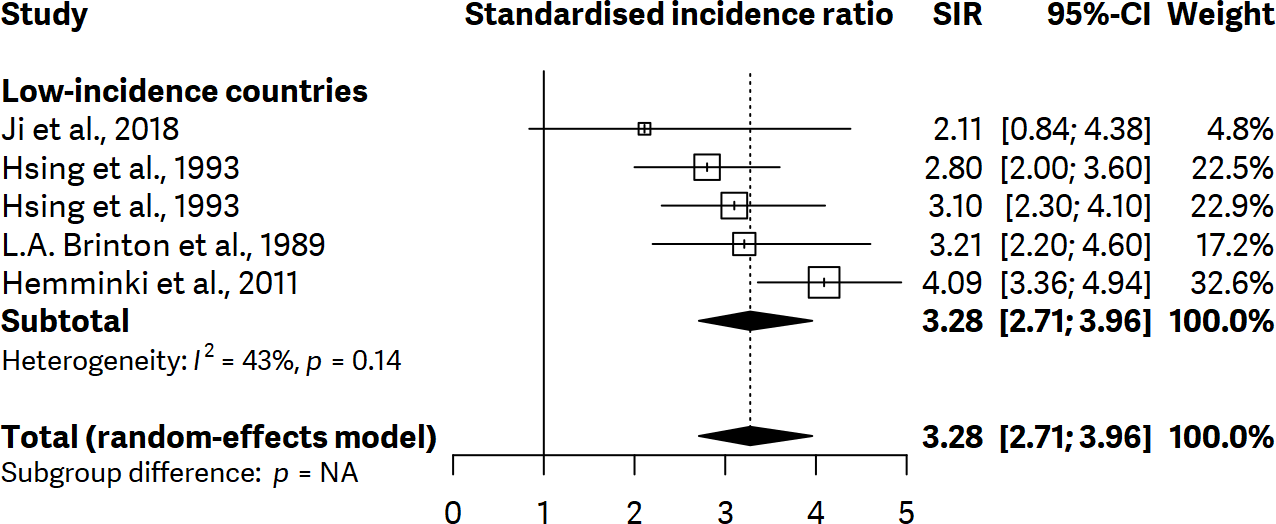
**

**Supplementary figure 50. Forest plot showing subgroup analysis regarding standardized incidence ratios (SIR) of gastric cancer with 95% confidence interval (CI) for inflammatory bowel disease based on high-, or low-incidence countries of gastric cancer.**

**
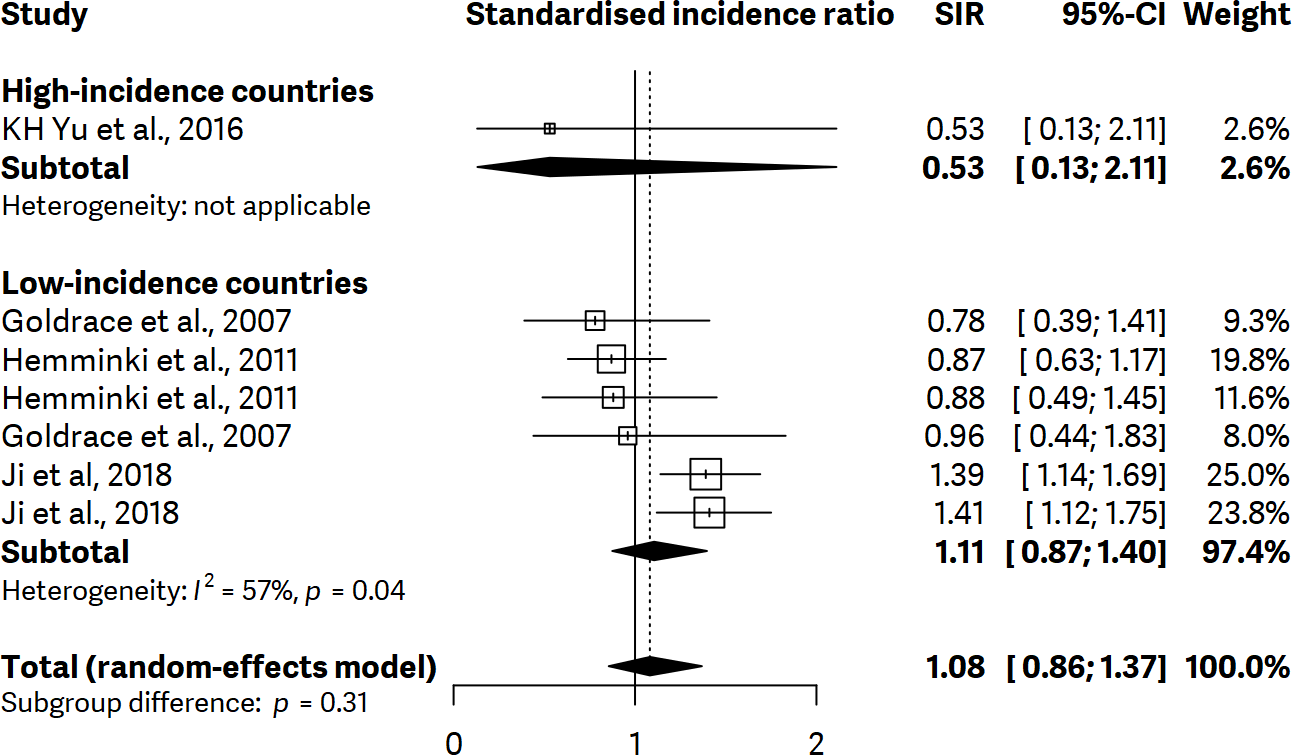
**

**Supplementary figure 51. Forest plot showing subgroup analysis regarding standardized incidence ratios (SIR) of gastric cancer with 95% confidence interval (CI) for inflammatory myopathies based on high-, or low-incidence countries of gastric cancer.**

**
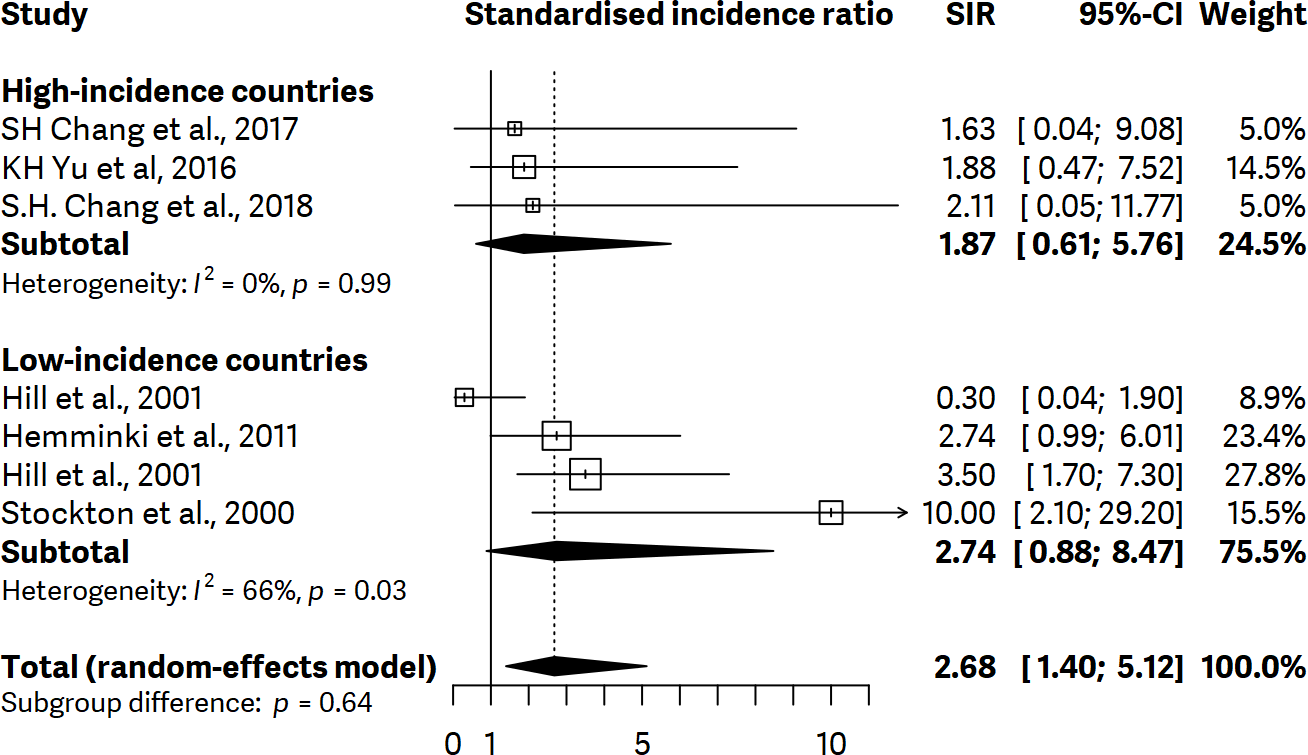
**

**Supplementary figure 52. Forest plot showing subgroup analysis regarding standardized incidence ratios (SIR) of gastric cancer with 95% confidence interval (CI) for primer biliary cirrhosis based on high-, or low-incidence countries of gastric cancer.**

**
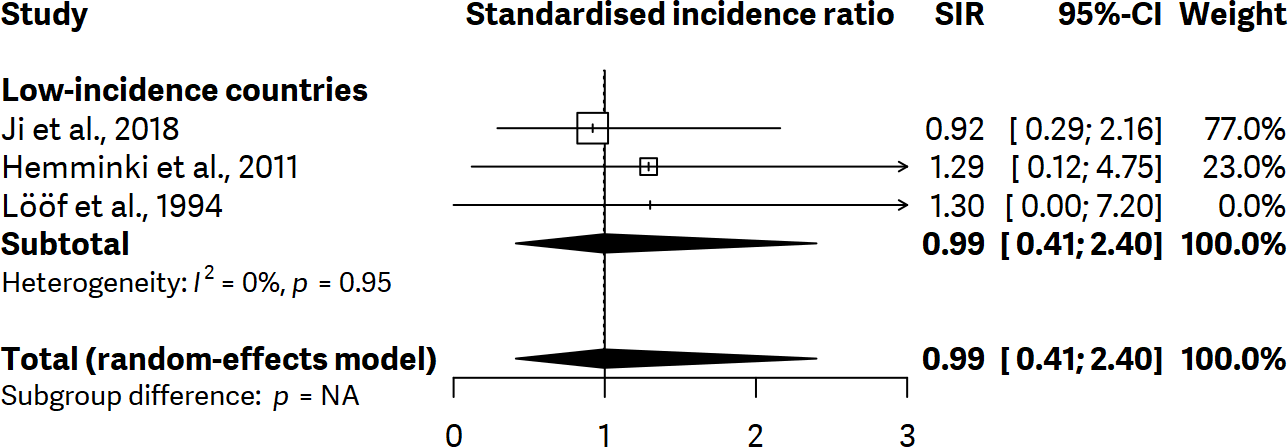
**

**Supplementary figure 53. Forest plot showing subgroup analysis regarding standardized incidence ratios (SIR) of gastric cancer with 95% confidence interval (CI) for rheumatoid arthritis based on high-, or low-incidence countries of gastric cancer.**

**
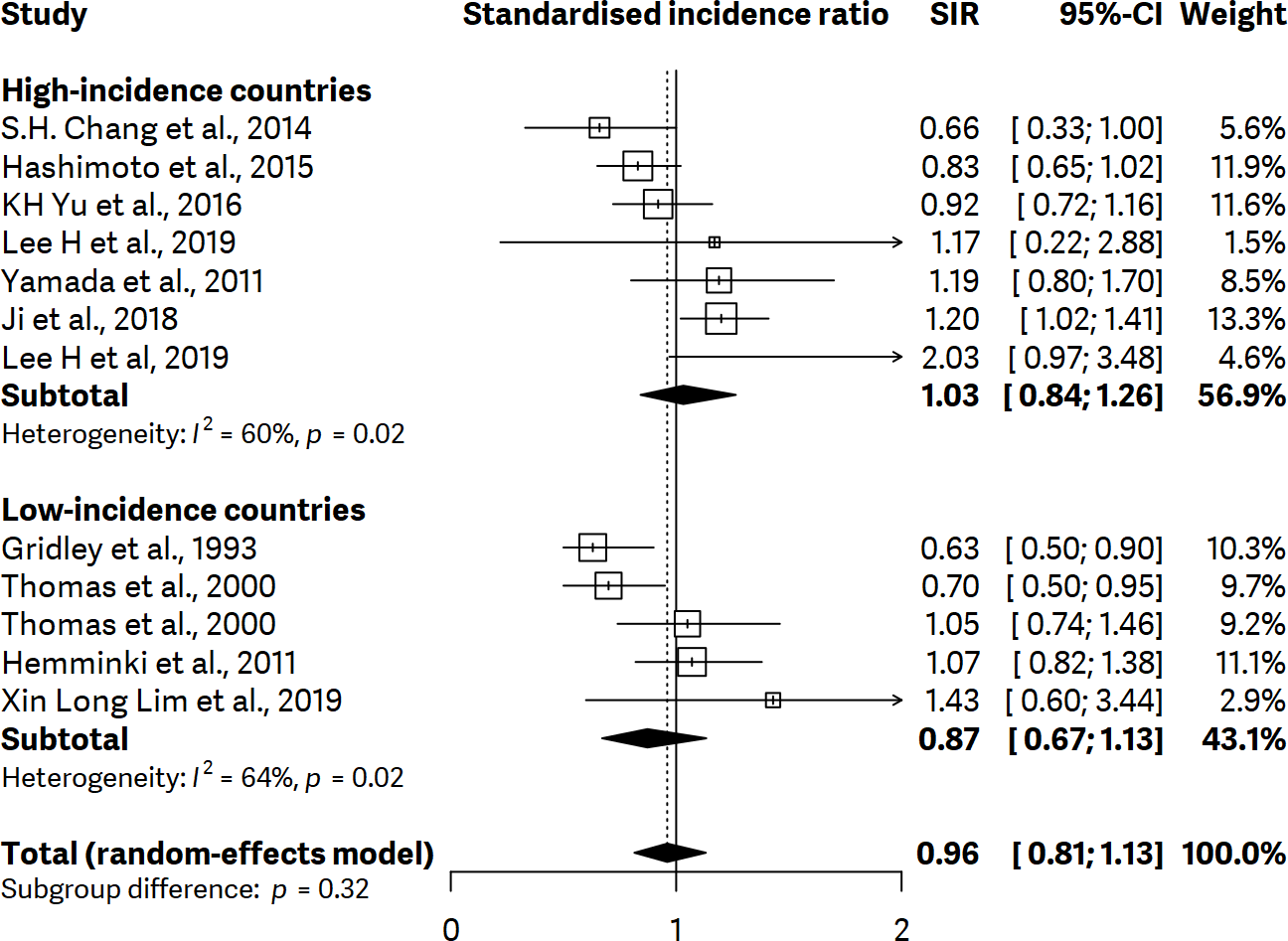
**

**Supplementary figure 54. Forest plot showing subgroup analysis regarding standardized incidence ratios (SIR) of gastric cancer with 95% confidence interval (CI) for Sjogren’s syndrome based on high-, or low-incidence countries of gastric cancer.**

**
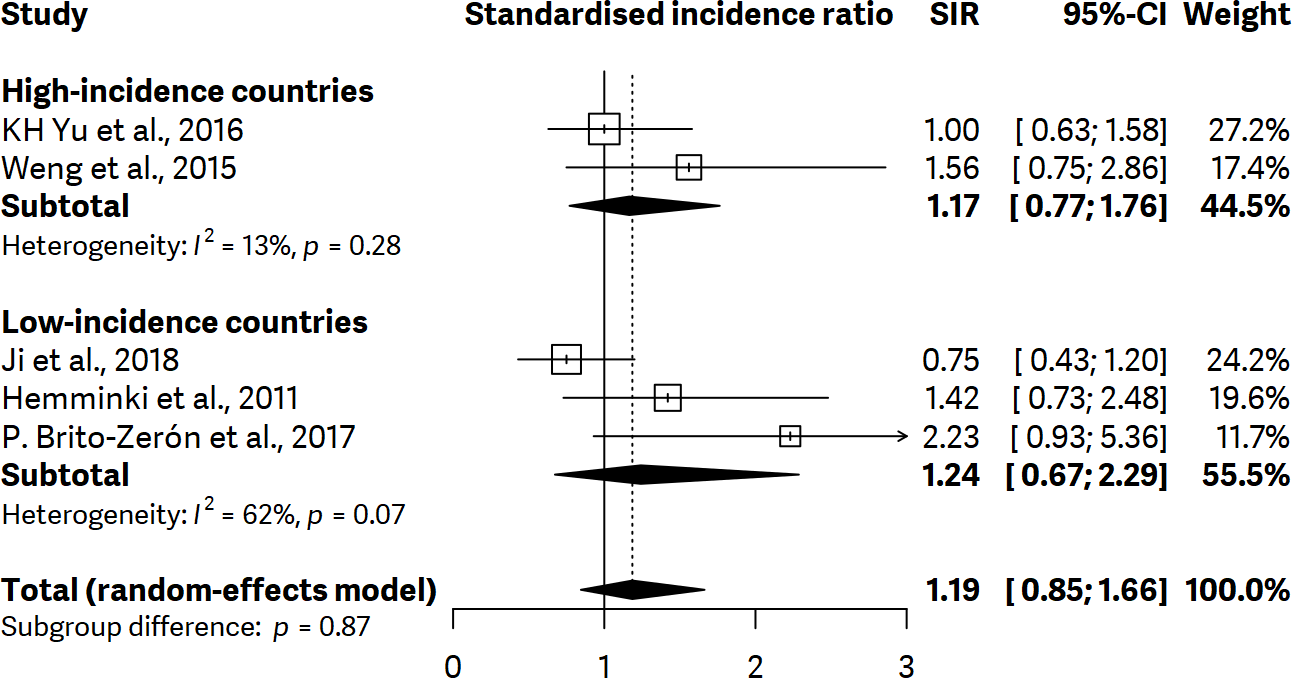
**

**Supplementary figure 55. Forest plot showing subgroup analysis regarding standardized incidence ratios (SIR) of gastric cancer with 95% confidence interval (CI) for systemic lupus erythematosus based on high-, or low-incidence countries of gastric cancer.**

**
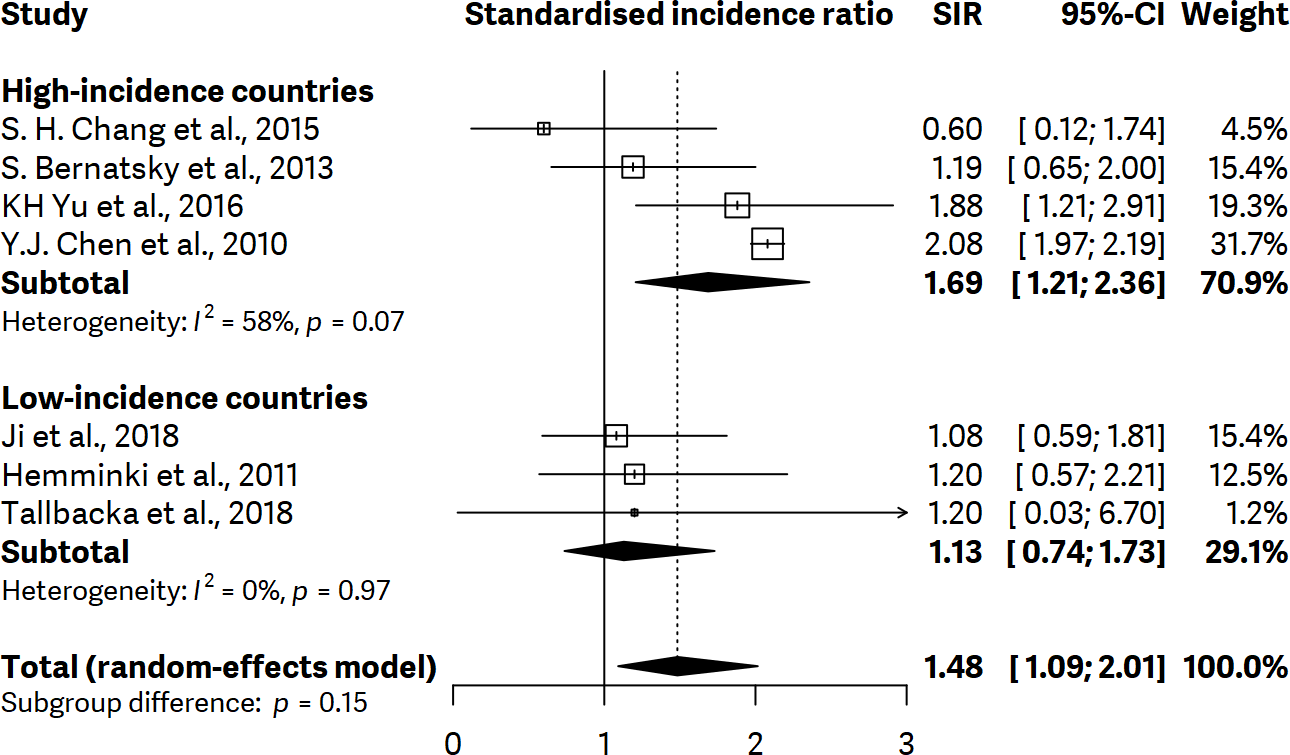
**

**Supplementary figure 56. Forest plot showing subgroup analysis regarding standardized incidence ratios (SIR) of gastric cancer with 95% confidence interval (CI) for systemic sclerosis based on high-, or low-incidence countries of gastric cancer.**

**
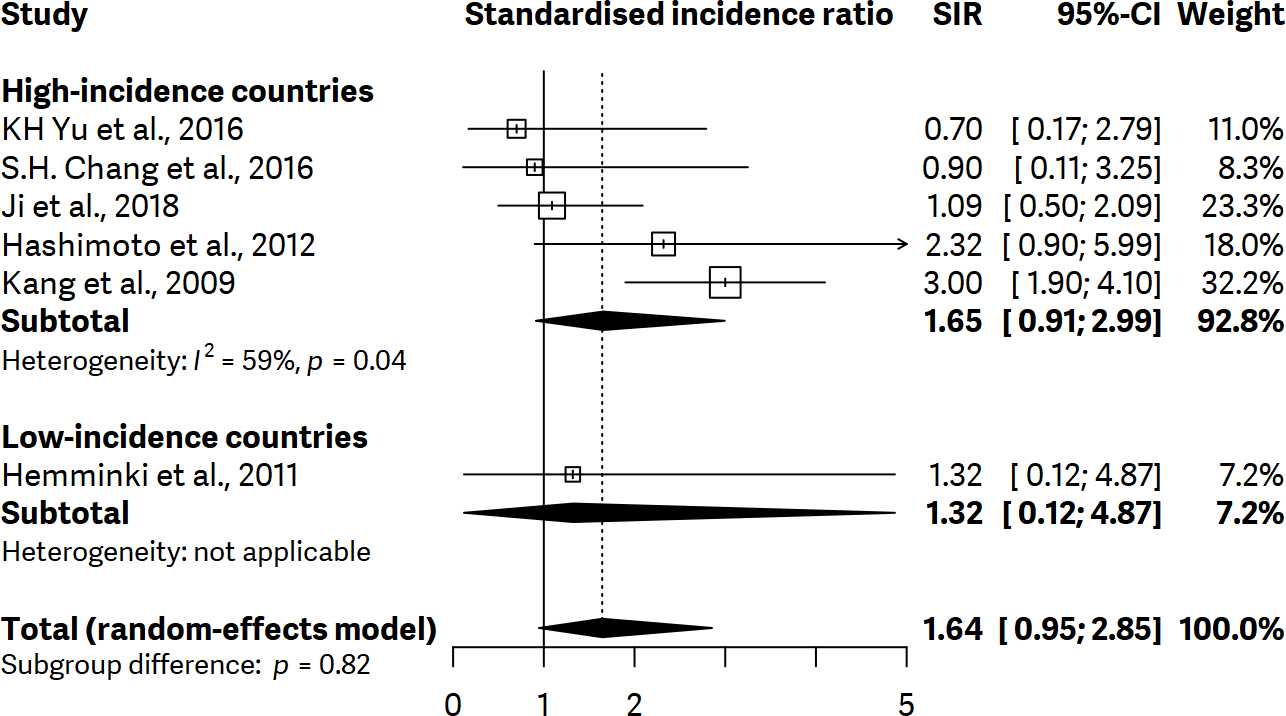
**

**Supplementary figure 57. Funnel plot for rheumatoid arthritis.**

**
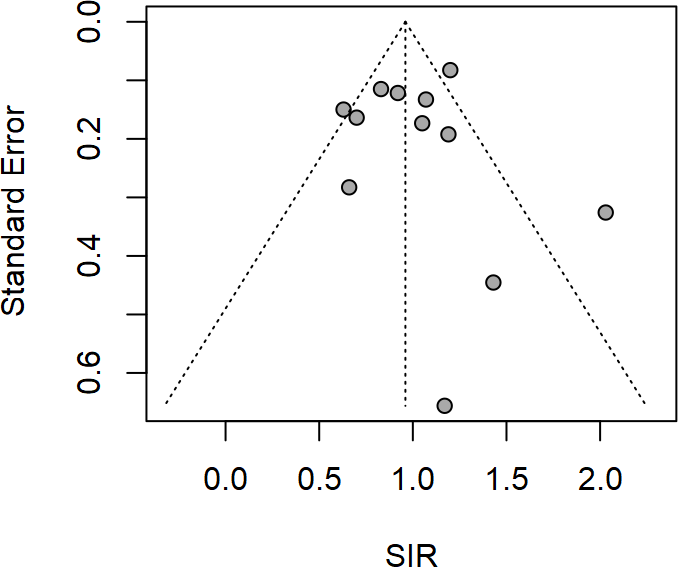
**

**References**

1. Page MJ, McKenzie JE, Bossuyt PM, et al. The PRISMA 2020 statement: an updated guideline for reporting systematic reviews. BMJ 2021;372:n71.

2. Hayden JA, van der Windt DA, Cartwright JL, Côté P, Bombardier C. Assessing bias in studies of prognostic factors. Annals of internal medicine 2013;158:280-286.

3. Asano J, Watanabe T, Oguchi T, et al. Association between immunoglobulin G4–related disease and malignancy within 12 Years after diagnosis: an analysis after longterm followup. The Journal of rheumatology 2015;42:2135-2142.

4. Askling J, Linet M, Gridley G, Halstensen TS, Ekström K, Ekbom A. Cancer incidence in a population-based cohort of individuals hospitalized with celiac disease or dermatitis herpetiformis. Gastroenterology 2002;123:1428-1435.

5. Bernatsky S, Ramsey-Goldman R, Labrecque J, et al. Cancer risk in systemic lupus: an updated international multi-centre cohort study. Journal of autoimmunity 2013;42:130-135.

6. Bjørneklett R, Vikse BE, Svarstad E, et al. Long-term risk of cancer in membranous nephropathy patients. American journal of kidney diseases 2007;50:396-403.

7. Brinton L, Gridley G, Hrubec Z, Hoover R, Fraumeni J. Cancer risk following pernicious anaemia. British journal of cancer 1989;59:810-813.

8. Brito-Zerón P, Kostov B, Fraile G, et al. Characterization and risk estimate of cancer in patients with primary Sjögren syndrome. Journal of hematology & oncology 2017;10:1-12.

9. Chang SH, Park JK, Lee YJ, et al. Comparison of cancer incidence among patients with rheumatic disease: a retrospective cohort study. Arthritis research & therapy 2014;16:1-6.

10. Chen Y-K, Lin C-L, Chang Y-J, et al. Cancer risk in patients with Graves' disease: a nationwide cohort study. Thyroid 2013;23:879-884.

11. Collin P, Pukkala E, Reunala T. Malignancy and survival in dermatitis herpetiformis: a comparison with coeliac disease. Gut 1996;38:528-530.

12. Dreyer L, Faurschou M, Mogensen M, Jacobsen S. High incidence of potentially virus‐induced malignancies in systemic lupus erythematosus: a long‐term followup study in a Danish cohort. Arthritis & Rheumatism 2011;63:3032-3037.

13. Goldacre MJ, Wotton CJ, Yeates D, Seagroatt V, Collier J. Liver cirrhosis, other liver diseases, pancreatitis and subsequent cancer: record linkage study. European journal of gastroenterology & hepatology 2008;20:384-392.

14. Gridley G, McLaughlin JK, Ekbom A, et al. Incidence of cancer among patients with rheumatoid arthritis. JNCI: Journal of the National Cancer Institute 1993;85:307-311.

15. Harding JL, Shaw JE, Peeters A, Cartensen B, Magliano DJ. Cancer risk among people with type 1 and type 2 diabetes: disentangling true associations, detection bias, and reverse causation. Diabetes care 2015;38:264-270.

16. Hashimoto A, Arinuma Y, Nagai T, et al. Incidence and the risk factor of malignancy in Japanese patients with systemic sclerosis. Internal Medicine 2012;51:1683-1688.

17. Hashimoto A, Chiba N, Tsuno H, et al. Incidence of malignancy and the risk of lymphoma in Japanese patients with rheumatoid arthritis compared to the general population. The Journal of rheumatology 2015;42:564-571.

18. Hemminki K, Liu X, Ji J, Sundquist J, Sundquist K. Autoimmune disease and subsequent digestive tract cancer by histology. Annals of oncology 2012;23:927-933.

19. Hill CL, Zhang Y, Sigurgeirsson B, et al. Frequency of specific cancer types in dermatomyositis and polymyositis: a population-based study. The Lancet 2001;357:96-100.

20. Hirano K, Tada M, Sasahira N, et al. Incidence of malignancies in patients with IgG4-related disease. Internal Medicine 2014;53:171-176.

21. Hsing AW, Hansson LE, McLaughlin JK, et al. Pernicious anemia and subsequent cancer. A population‐based cohort study. Cancer 1993;71:745-750.

22. Hsu P-C, Lin W-H, Kuo T-H, Lee H-M, Kuo C, Li C-Y. A population-based cohort study of all-cause and site-specific cancer incidence among patients with type 1 diabetes mellitus in Taiwan. Journal of epidemiology 2015:JE20140197.

23. Ilus T, Kaukinen K, Virta LJ, Pukkala E, Collin P. Incidence of malignancies in diagnosed celiac patients: a population-based estimate. Official journal of the American College of Gastroenterology| ACG 2014;109:1471-1477.

24. Ji J, Sundquist J, Sundquist K. Family history of autoimmune diseases and risk of gastric cancer: a national cohort study. European Journal of Cancer Prevention 2018;27:221-226.

25. Yu K-H, Kuo C-F, Huang LH, Huang W-K, See L-C. Cancer risk in patients with inflammatory systemic autoimmune rheumatic diseases: a nationwide population-based dynamic cohort study in Taiwan. Medicine 2016;95.

26. Kang K, Yim H, Kim IJ, et al. Incidence of cancer among patients with systemic sclerosis in Korea: results from a single centre. Scandinavian journal of rheumatology 2009;38:299-303.

27. Kirkegård J, Farkas DK, Jørgensen JOL, Cronin-Fenton DP. Hyperthyroidism or hypothyroidism and gastrointestinal cancer risk: a Danish nationwide cohort study. Endocrine connections 2018;7:1129-1135.

28. Koskinen I, Hervonen K, Pukkala E, Reunala T, Kaukinen K, Collin P. Cancer incidence and factors associated with malignancies in coeliac disease during long‐term follow‐up. GastroHep 2021;3:107-115.

29. Lee H. The risk of malignancy in korean patients with rheumatoid arthritis. Yonsei medical journal 2019;60:223-229.

30. Lööf L, Adami HO, Sparén P, et al. Cancer risk in primary biliary cirrhosis: a population‐based study from Sweden. Hepatology 1994;20:101-104.

31. Nam B, Kim H, Jang EJ, Cho S-K, Sung Y-K, Kim T-H. Malignancy risk in Korean male patients with ankylosing spondylitis. Rheumatology international 2019;39:1741-1748.

32. Park JK, Choi IA, Lee EY, Song YW, Lee EB. Incidence of malignancy in Takayasu arteritis in Korea. Rheumatology international 2014;34:517-521.

33. Shiokawa M, Kodama Y, Yoshimura K, et al. Risk of cancer in patients with autoimmune pancreatitis. Official journal of the American College of Gastroenterology| ACG 2013;108:610-617.

34. Shu X, Ji J, Li X, Sundquist J, Sundquist K, Hemminki K. Cancer risk among patients hospitalized for Type 1 diabetes mellitus: a population‐based cohort study in Sweden. Diabetic medicine 2010;27:791-797.

35. Silano M, Volta U, Mecchia AM, Dessì M, Di Benedetto R, De Vincenzi M. Delayed diagnosis of coeliac disease increases cancer risk. BMC gastroenterology 2007;7:1-5.

36. Stockton D, Doherty V, Brewster D. Risk of cancer in patients with dermatomyositis or polymyositis, and follow-up implications: a Scottish population-based cohort study. British journal of cancer 2001;85:41-45.

37. Swerdlow A, Laing S, Qiao Z, et al. Cancer incidence and mortality in patients with insulin-treated diabetes: a UK cohort study. British journal of cancer 2005;92:2070-2075.

38. Tallbacka K, Pettersson T, Pukkala E. Increased incidence of cancer in systemic lupus erythematosus: a Finnish cohort study with more than 25 years of follow-up. Scandinavian journal of rheumatology 2018;47:461-464.

39. Thomas E, Brewster DH, Black RJ, Macfarlane GJ. Risk of malignancy among patients with rheumatic conditions. International journal of cancer 2000;88:497-502.

40. van Daalen EE, Rahmattulla C, Wolterbeek R, Bruijn JA, Bajema IM. Incidence of Malignancy Prior to Antineutrophil Cytoplasmic Antibody–associated Vasculitis Compared to the General Population. The Journal of rheumatology 2017;44:314-318.

41. Viljamaa M, Kaukinen K, Pukkala E, Hervonen K, Reunala T, Collin P. Malignancies and mortality in patients with coeliac disease and dermatitis herpetiformis: 30-year population-based study. Digestive and Liver Disease 2006;38:374-380.

42. Weng M-Y, Huang Y-T, Liu M-F, Lu T-H. Incidence of cancer in a nationwide population cohort of 7852 patients with primary Sjögren's syndrome in Taiwan. Annals of the rheumatic diseases 2012;71:524-527.

43. Lim XR, Xiang W, Tan JWL, et al. Incidence and patterns of malignancies in a multi‐ethnic cohort of rheumatoid arthritis patients. International journal of rheumatic diseases 2019;22:1679-1685.

44. Yamada T, Nakajima A, Inoue E, et al. Incidence of malignancy in Japanese patients with rheumatoid arthritis. Rheumatology international 2011;31:1487-1492.

45. Yoo J, Ahn SS, Jung SM, Song JJ, Park Y-B, Lee S-W. Cancer development in Korean patients with ANCA-associated vasculitis: a single centre study. Clin Exp Rheumatol 2018;36:73-77.
